# A Modern Genotoxicity Testing Paradigm: Integration of the High-Throughput CometChip® and the TGx-DDI Transcriptomic Biomarker in Human HepaRG™ Cell Cultures

**DOI:** 10.3389/fpubh.2021.694834

**Published:** 2021-08-18

**Authors:** Julie K. Buick, Andrew Williams, Matthew J. Meier, Carol D. Swartz, Leslie Recio, Rémi Gagné, Stephen S. Ferguson, Bevin P. Engelward, Carole L. Yauk

**Affiliations:** ^1^Environmental Health Science and Research Bureau, Health Canada, Ottawa, ON, Canada; ^2^Integrated Laboratory Systems Inc. (ILS), Research Triangle Park, Durham, NC, United States; ^3^National Toxicology Program, National Institute of Environmental Health Sciences, Research Triangle Park, Durham, NC, United States; ^4^Department of Biological Engineering, Massachusetts Institute of Technology, Cambridge, MA, United States; ^5^Department of Biology, University of Ottawa, Ottawa, ON, Canada

**Keywords:** genetic toxicology, TGx-DDI genomic biomarker, TGx-28.65 genomic biomarker, metabolic activation, toxicogenomics, human health risk assessment

## Abstract

Higher-throughput, mode-of-action-based assays provide a valuable approach to expedite chemical evaluation for human health risk assessment. In this study, we combined the high-throughput alkaline DNA damage-sensing CometChip^®^ assay with the TGx-DDI transcriptomic biomarker (DDI = DNA damage-inducing) using high-throughput TempO-Seq^®^, as an integrated genotoxicity testing approach. We used metabolically competent differentiated human HepaRG™ cell cultures to enable the identification of chemicals that require bioactivation to cause genotoxicity. We studied 12 chemicals (nine DDI, three non-DDI) in increasing concentrations to measure and classify chemicals based on their ability to damage DNA. The CometChip^®^ classified 10/12 test chemicals correctly, missing a positive DDI call for aflatoxin B1 and propyl gallate. The poor detection of aflatoxin B1 adducts is consistent with the insensitivity of the standard alkaline comet assay to bulky lesions (a shortcoming that can be overcome by trapping repair intermediates). The TGx-DDI biomarker accurately classified 10/12 agents. TGx-DDI correctly identified aflatoxin B1 as DDI, demonstrating efficacy for combined used of these complementary methodologies. Zidovudine, a known DDI chemical, was misclassified as it inhibits transcription, which prevents measurable changes in gene expression. Eugenol, a non-DDI chemical known to render misleading positive results at high concentrations, was classified as DDI at the highest concentration tested. When combined, the CometChip^®^ assay and the TGx-DDI biomarker were 100% accurate in identifying chemicals that induce DNA damage. Quantitative benchmark concentration (BMC) modeling was applied to evaluate chemical potencies for both assays. The BMCs for the CometChip^®^ assay and the TGx-DDI biomarker were highly concordant (within 4-fold) and resulted in identical potency rankings. These results demonstrate that these two assays can be integrated for efficient identification and potency ranking of DNA damaging agents in HepaRG™ cell cultures.

## Introduction

New tools and approaches are urgently needed to allow regulatory agencies worldwide to evaluate a backlog of chemicals for potential adverse human health effects (1–7). Twenty-first century toxicology requires more affordable tests that are higher-throughput, higher-content, human-relevant, and mechanistic in nature for effective chemical evaluation (8–13). Applying *in vitro* toxicogenomic (TGx) biomarkers in metabolically competent human cells in culture is a new approach methodology (NAM) that can help to accomplish these goals. Transcriptomic biomarkers are defined gene sets that produce reproducible changes for altered key events in adverse outcome pathways. These biomarkers can be used to identify chemical mode of action (MoA) and to guide chemical prioritization and classification (14–19). The use of *in vitro* genomic biomarkers to predict specific toxicological responses reduces the subjectivity of interpretation for complex genomic data sets and can thus facilitate the use of genomics for human health risk assessment.

Genetic damage can lead to mutagenicity and genome instability, which in turn can result in adverse human health effects, such as inherited genetic diseases and cancer (20). Consequently, genotoxicity testing is a critical component of chemical evaluation. Genotoxicity and mutagenicity testing depends on jurisdiction, but generally includes the Ames bacterial reverse mutation assay, an *in vitro* mammalian genotoxicity assay (e.g., chromosome aberrations, micronuclei (MN), and/or gene mutations), and an *in vivo* rodent genotoxicity assay [e.g., chromosome aberrations, MN, and/or transgene mutations; (21–24)]. These tests are not typically high-throughput and generally do not provide mechanistic insight into a test compound's MoA. Higher-throughput, mechanism-based genotoxicity tests in human cell culture models can aid in the interpretation of these assays to determine potential human risk. More recent advances to modernize genetic toxicity assays have begun to address this need; for example, the *in vitro* MicroFlow^®^ micronucleus assay (25–28) and the *in vitro* CometChip^®^ assay (29–33) are compatible with various human and rodent cell lines and are relatively higher-throughput methods to assess DNA damage.

A long-term goal is to have a suite of transcriptomic biomarkers that enable rapid extraction of MoA and hazard information from high-throughput transcriptomic (HTTr) screens (7). The TGx-DDI biomarker provides an alternative approach wherein potentially genotoxic MoAs can be discerned using transcriptomic data sets. The TGx-DDI transcriptomic biomarker was developed from a training set of global gene expression profiles from human TK6 cells exposed to 28 prototype DNA damage-inducing (DDI) or non-DDI chemicals (34–37). The biomarker comprises 64 genes; changes in the expression of these genes can be used to classify chemicals as DDI or non-DDI using a variety of gene expression technologies in TK6 cells, including DNA microarrays (35), quantitative PCR arrays (38) and the high-throughput NanoString nCounter^®^ platform (36). Overall, it has been proposed that the TGx-DDI biomarker can be used in a variety of contexts including chemical screening (39), hazard identification, chemical prioritization for further testing, MoA development, weight of evidence analysis, and/or potency assessment (36).

Currently, tremendous efforts are being made to develop suitable cell-based assays as a reliable and informative substitute for *in vivo* studies. Although, human TK6 cells are a suitable choice of cell line to evaluate genotoxicity for regulatory applications, a substantial limitation is that they lack metabolic activity. Primary human hepatocytes, often considered the gold standard for physiologically-relevant *in vitro* liver cell culture models, also have some notable limitations in that they have a finite supply from an individual donor making them difficult to obtain in large quantities for year-over-year evaluations, have a highly limited lifespan of differentiated functionality in conventional 2D culture models (~3–5 days), and in some countries are not an ethically viable option (40–42). A suitable alternative is to use human HepaRG™ cells, which were derived from a hepatocellular carcinoma in a Caucasian female donor (43). Under differentiating conditions, HepaRG™ cell cultures express relevant amounts of Phase I and Phase II metabolic enzymes, transporters and nuclear receptors, and differentiate into co-cultures of hepatocyte- and cholangiocyte-like cells, which makes them a suitable choice for toxicity screening (43). HepaRG™ cells retain many characteristics of primary human hepatocytes and thus circumvent the need to add rat liver S9, which can be problematic for some compounds and is a limitation of the TK6 cell line. Moreover, HepaRG™ cells have undergone extensive validation for *in vitro* cytochrome P450 induction and have been deemed a reliable human cell line in terms of metabolic competence (44–51). This was further confirmed by an interlaboratory validation of liver enzyme induction models led by the European Commission, Joint Research Center (52). The popularity of these cells in toxicology studies is thus growing, and they are currently being used in HTTr screens. For example, Ramaiahgari et al. (53) used high-throughput targeted RNA-sequencing (TempO-Seq^®^; Templated Oligo-Sequencing) in HepaRG™ cells to conduct concentration-response modeling for 24 reference compounds to explore transcriptomic characteristics distinguishing compounds that result in drug-induced liver injury.

The use of the TGx-DDI genomic biomarker in metabolically competent human HepaRG™ cell culture is advantageous. Our pilot work showed that the biomarker was 100% accurate in identifying five DDI and five non-DDI toxicants in HepaRG™ cells by RNA-sequencing (54). Corton et al. (39) also demonstrated predictive accuracies of 90% in identifying DDI agents in HepaRG™ cells using the TGx-DDI biomarker in combination with a pattern matching correlation approach. Nevertheless, additional validation studies that confirm the accuracy of the TGx-DDI biomarker in human HepaRG™ cells using the most recent HTTr platforms would be tremendously useful to advance its application in such chemical screens for genotoxic hazard identification.

The comet assay offers an alternative approach to genotoxicity testing that directly tests for the presence of physical damage to DNA. We reasoned that together, these two approaches would be highly complementary, providing an efficient integrated test to accurately identify genotoxic agents. While the traditional comet assay is not compatible with high-throughput screens due to the need for a single glass slide for each condition, the recently available high-throughput CometChip^®^ platform has >200x the capacity of the traditional comet assay for identifying chemicals that induce DNA strand breaks and has been extensively validated (30–33, 55, 56). The objectives of the present study are thus to: (1) extend validation efforts of the TGx-DDI genomic biomarker further through analysis of HepaRG™ cells exposed to prototype DDI and non-DDI agents; (2) confirm the predictive accuracy of TGx-DDI using the TempO-Seq^®^ platform; (3) explore the integration of the high-throughput alkaline CometChip^®^ assay and the TGx-DDI biomarker as an efficient, next-generation genotoxicity screening approach to identify DDI chemicals; and (4) conduct concentration-response modeling to investigate chemical potency ranking for DNA damage measured by the CometChip^®^ assay vs. transcriptional changes in the TGx-DDI biomarker genes.

Herein we investigate 12 test chemicals with varied MoAs, chemical uses, and effects (Table 1). For practical purposes, we classified the test chemicals as either DDI or non-DDI. All DDI test compounds are Group 1 chemicals based on the Kirkland et al. (57, 58) recommended lists of genotoxic chemicals for the assessment of the performance of new or improved genotoxicity tests and should render a positive result in mammalian genotoxicity tests in culture. In this study, the DDI chemicals include: aflatoxin B1 (AFB1), benzo[a]pyrene (BaP), cisplatin (CISP), cyclophosphamide (CP), cytosine arabinoside (AraC), methyl methanesulfonate (MMS), N-nitroso-N-ethylurea (ENU), and zidovudine (ZDV; also known as azidothymidine). The non-DDI group of chemicals in this study can be further broken down into one well-established non-DDI chemical (i.e., Group 2) and three potentially misleading (irrelevant) positives (i.e., Group 3) based on the Kirkland et al. (57, 58) recommended list of non-genotoxic chemicals. In this study, 2-deoxy-D-glucose (2DG) is a non-DDI Group 2 chemical based on the criteria presented in the Kirkland et al. (57, 58) reports, as 2DG is expected to render a negative result in *in vitro* human and rodent cell-based genotoxicity tests. Group 3 chemicals should also yield negative results in genotoxicity tests with mammalian cells. Chemicals in Group 3 are most often negative in the Ames assay and *in vivo*; however, chemicals in this grouping have been reported to induce DNA damage, most often at high concentrations or with high levels of cytotoxicity, which leads to “misleading” positive results. Based on the Kirkland et al. (57) recommended lists, eugenol (EUG), propyl gallate (PG), and urea were all considered to be Group 3 chemicals that have the potential to result in misleading positive results. However, based on the updated recommended chemical list published by Kirkland et al. (58), PG is reported as positive in the Ames test with S9 and induces MN and chromosomal aberrations *in vivo* (59, 60). Thus, PG has now been removed from Group 3, as it is potentially DNA-reactive and positive *in vivo* for certain genotoxic endpoints. Although, PG is not a Group 1 reference chemical, we expect it to classify as a Group 1; hence, we have grouped PG with the DDI chemicals herein.

**Table 1:** Test chemical information for Group 1 (genotoxic/DDI chemicals), Group 2 (non-genotoxic/non-DDI chemicals), and Group 3 (misleading/irrelevant positive chemicals) based on the recommended genotoxic and non-genotoxic chemicals for assessment of the performance of new or improved genotoxicity tests by Kirkland et al. (57, 58).

	**Chemical use/formation**	**Chemical effects**	**Kirkland et al. (57, 58) chemical group**
Aflatoxin B1	Food contaminant produced by pathogenic fungus	Forms DNA adducts; clastogenic, mutagenic, teratogenic, carcinogenic	Group 1
Benzo[a]pyrene	Polycyclic aromatic hydrocarbon; formed during incomplete combustion	Forms DNA adducts; clastogenic, mutagenic, carcinogenic	Group 1
Cisplatin	Chemotherapeutic agent	Alkylating agent that interferes with DNA replication; cross-linking agent; clastogenic and mutagenic	Group 1
Cyclophosphamide	Chemotherapeutic agent	Alkylating agent; clastogenic, mutagenic	Group 1
Cytosine arabinoside	Chemotherapeutic agent	DNA anti-metabolite that interferes with DNA replication; clastogenic	Group 1
Methyl methanesulfonate	Chemotherapeutic agent	Alkylating agent; clastogenic, mutagenic, carcinogenic	Group 1
N-Ethyl-N-nitrosourea	Chemotherapeutic agent	Alkylating agent; clastogenic, mutagenic, carcinogenic and teratogenic	Group 1
Zidovudine (Azidothymidine)	Anti-HIV drug	Nucleoside reverse transcriptase inhibitor (NRTI); clastogenic	Group 1
Propyl gallate	Antioxidant; food additive	Used to prevent oxidation; mutagenic and clastogenic	(Group 1)^#^
2-Deoxy-D-glucose	Used as a diagnostic agent in its radiolabelled form	Investigational drug that is being studied as an anticancer and antiviral agent; glycolysis inhibitor	Group 2^*^
Eugenol	Naturally occurring phenolic molecule found in plants; local analgesic agent to alleviate tooth pain	Interferes with action potential conduction; has anti-inflammatory, neuroprotective, antipyretic, antioxidant, antifungal and analgesic properties	Group 3
Urea	Organic compound important in the metabolism of nitrogen-containing compounds by animals	Nitrogen-containing substance in mammalian urine, also used in fertilizers; non-toxic	Group 3

# *PG was removed from Group 3 in the Kirkland et al. (58) updated recommended lists, as PG is now reported to be positive in the Ames test in the presence of S9 and induces micronuclei and chromosomal aberrations in vivo. We have thus included PG as Group 1*.

* *Chemical not included in Kirkland et al. (57, 58) recommended lists for non-genotoxic chemical, but 2-deoxy-D-glucose fits the criteria to be included in Group 2 (non-genotoxic chemical) and is used as a non-DDI reference chemical in the development of the TGx-DDI biomarker in Li et al. (35)*.

Taken together, we present results for nine DDI, one non-DDI and two potentially misleading DDI agents. We found that combining a DNA damage assay that rapidly detects DNA strand breaks (i.e., the CometChip^®^) with the TGx-DDI genomic biomarker in metabolically competent HepaRG™ cells provides and efficient and accurate approach to identify and rank potencies of chemicals.

## Materials and Methods

### Chemicals

Test chemicals were purchased from Cayman Chemical (CISP; Ann Arbor, MI, USA), TCI America (PG; Montgomeryville, PA, USA), and Millipore Sigma (remaining chemicals; St. Louis, MO, USA) for exposures in fully differentiated, cryopreserved No-Spin HepaRG™ cells (Triangle Research Labs (TRL), Durham, NC, USA; acquired by Lonza Bioscience). Test chemical information, including corresponding vehicle control and concentrations tested are shown in Table 2. The chemical exposures in HepaRG™ cells, the cell viability studies, and the paired high-throughput CometChip^®^ analysis were conducted at Integrated Laboratory Systems, Inc. (ILS; Research Triangle Park, Durham, NC, USA).

**Table 2:** Experimental information for DDI (genotoxic) and non-DDI (non-genotoxic) test chemicals used in this study.

**Test chemical**	**Chemical abbreviation**	**CAS No**.	**Chemical group^**#**^**	**Vehicle control**	**Concentrations tested (μm)**
Aflatoxin B1	AFB1	1162-65-8	Group 1	DMSO	3.125, 6.25, 12.5, 15, 25
Benzo[a]pyrene	BaP	50-32-8	Group 1	DMSO	0.9375, 1.875, 3.75, 7.5, 15
Cisplatin	CISP	15663-27-1	Group 1	DMSO	3.125, 6.25, 12.5, 25, 50
Cyclophosphamide	CP	6055-19-2	Group 1	DMSO	1,250, 2,500, 5,000, 7,500, 10,000
Cytosine arabinoside	AraC	147-94-4	Group 1	DMSO	12.5, 25, 50, 100, 200
Methyl methanesulfonate	MMS	66-27-3	Group 1	DMSO	22.7, 45.4, 90.8, 181.6, 363.2
N-Nitroso-N-ethylurea	ENU	759-73-9	Group 1	DMSO	312.5, 625, 1,250, 2,500, 5,000^*^
Zidovudine (azidothymidine)	ZDV	30516-87-1	Group 1	DMSO	125, 250, 500, 1,000, 2,000
Propyl gallate	PG	121-79-9	(Group 1)	DMSO	125, 250, 500, 750, 1,000^*^
2-Deoxy-D-glucose	2DG	154-17-6	Group 2	Water	625, 1,250, 2,500, 5,000, 10,000
Eugenol	EUG	97-53-0	Group 3	DMSO	156.25, 312.5, 625, 1,250, 2,500^*^
Urea	Urea	57-13-6	Group 3	DMSO	625, 1,250, 2,500, 5,000, 10,000

# *Chemical grouping based on Kirkland et al. (57, 58) recommended lists of genotoxic and non-genotoxic chemicals for assessment of the performance of new or improved genotoxicity tests*.

* *Indicates a cytotoxic concentration (<40% cell viability; >60% cytotoxic) that was subsequently eliminated from the gene expression analysis; all concentrations were used for CometChip^®^ analysis*.

### HepaRG™ Cell Culture and Chemical Exposures

Human HepaRG™ cell cultures were exposed to increasing concentrations of 12 test chemicals in parallel 96-well plates (four test chemicals per plate) for assessment of DNA damage by CometChip^®^ and for collection of cell lysates for TempO-Seq^®^ analysis for TGx-DDI classification purposes. Concentration setting for each test chemical was based on data previously collected at ILS and was established from the observation of either a robust positive CometChip^®^ response or an upper concentration that was approaching (but not above) an overt cytotoxicity threshold (<40% viable cells) in previous in-house studies. In the absence of a positive CometChip^®^ response or cytotoxicity, chemicals were tested up to a top concentration of 10 mM, which is compliant for non-cytotoxic, negative compounds in OECD test guidelines for mammalian cell assays (61, 62). Briefly, differentiated human HepaRG™ cells, derived from a hepatocellular carcinoma (45) were thawed and seeded at ~4.0–5.0 × 10^4^ viable cells per well in a collagen-coated 96-well CometChip^®^ in William's E medium with TRL's Thawing and Plating Supplement. Cells were incubated for 7 days following seeding to allow the cells to regain peak metabolic function (45). Cells were then exposed in culture medium containing TRL's Pre-Induction/Tox Supplement to five concentrations of each DDI or non-DDI chemical daily in a repeated exposure design (exposures at 0, 24, and 48 h). Four hours following the last treatment (52 h total time), one plate of cells was used for cell viability (*n* = 2 per treatment group alongside matched solvent controls) and CometChip^®^ analysis and the second plate was used to generate cell lysates for gene expression analysis (*n* = 4 per treatment group per assay for CometChip^®^ and TempO-Seq^®^). The media was aspirated from exposed cells and they were washed with PBS, prior to adding 100 μl of TrypLE™ (for cell viability and CometChip^®^ assays; ThermoFisher Scientific, Waltham, MA, USA) or 1X TempO-Seq^®^ Lysis Buffer in PBS (for TempO-Seq^®^ assay; BioSpyder Technologies, Carlsbad, CA, USA) to each well to lyse cells for 10 min at room temperature. Cell lysates were then frozen and stored at −80°C for subsequent transcriptome profiling described below. Samples used for the analysis of cell viability and DNA damage using CometChip^®^ were neutralized with the addition of 100 μl of culture medium to each well and were processed as described in the following sections.

### Cell Viability Assay

The CellTiter-Glo^®^ Luminescent Cell Viability Assay (Promega, Madison, WI, USA) was used to determine the number of viable HepaRG™ cells based on the quantification of ATP present following each chemical treatment. Cytotoxicity was evaluated 4 h after the last exposure following the manufacturer's instructions in 96-well plates. Briefly, wells containing 100 μl cell samples were equilibrated at room temperature for 30 min prior to the addition of CellTiter-Glo^®^ Reagent to each well in a volume equal to that of the cell culture medium (e.g., 100 μl). The contents were mixed for 2 min on an orbital shaker to induce cell lysis prior to incubation at room temperature for 10 min to stabilize the luminescent signal. Luminescence was measured on a SpectraMax^®^ plate reader (Molecular Devices, San Jose, CA, USA). Luminescent signal is the result of the release of ATP from metabolically active cells and is directly proportional to the number of viable cells in the culture. The cytotoxicity cut-off was >60% cytotoxic (equivalent to <40% viable cells).

### Trevigen CometChip® Assay

Exposed and control HepaRG™ cells were loaded into the CometChip^®^ wells and were allowed to settle into microwells of a 96-well CometChip^®^. A 1% agarose overlay was then applied and the cells were lysed in cold lysis buffer (2.5 M NaCl, 100 mM EDTA, 10 mM Tris, pH 10 with 1% Triton X-100 (Sigma, St. Louis, MO, USA) and 10% DMSO) overnight at 4°C. Following lysis, the CometChip^®^ was equilibrated in an alkaline electrophoresis buffer (300 mM NaOH/1 mM EDTA) for 40 min and electrophoresed for 50 min under a 300 mA current at 4°C. These alkaline conditions are used to detect DNA single strand breaks (SSBs). Following electrophoresis, the CometChip^®^ was neutralized at 4°C for 2 × 15 min in 0.4 M Tris, pH 7.4 and equilibrated overnight at 4°C in 20 mM Tris, pH 7.4. Once equilibrated, the chip was stained for 30 min at 4°C in 0.1X SYBR Gold and then destained for >1 h at 4°C in 20 mM Tris, pH 7.4. After destaining, images were taken at 4X magnification of all 96 wells. The tiff images were captured and analyzed using Trevigen^®^ Comet Analysis Software.

### Statistical Analysis of CometChip® Data

The median % tail DNA for the CometChip^®^ data was analyzed using one-way analysis of variance (ANOVA). The Anderson-Darling statistic was used to test the normality assumption and the Fligner-Killeen test of homogeneity of variances was used to test the common variance assumption. If either assumption was not satisfied, the rank transformation was applied and the non-parametric one-way ANOVA was performed (63). All pairwise comparisons to matched vehicle controls were conducted using the *t*-test. The resulting *p*-values were then FWER adjusted using the Dunnett's method.

### TempO-Seq® Library Preparation and S1500+ Targeted Transcriptome Sequencing

The TempO-Seq^®^ Human Tox+Surrogate Panel Reagent Kit (BioSpyder Technologies, Carlsbad, CA, USA) was used to prepare libraries in a 96-well plate format from exposed and control HepaRG™ cell lysates, according to the manufacturer's instructions. All five concentrations of each test chemical were used for gene expression analysis, except for overtly cytotoxic concentrations that were eliminated from the analysis [i.e., the highest concentration (C5) of ENU, EUG and PG; Table 2]. Assay controls included a negative no-lysate control (1X TempO-Seq^®^ Lysis Buffer only), and two positive controls: qPCR Human Reference Total RNA and Human Brain Total RNA (Takara Bio, CA, USA; four replicates per control). Briefly, 2 μl of cell lysate in 1X TempO-Seq^®^ Lysis Buffer from each treatment and concentration were hybridized with the targeted Human S1500+ Tox Panel detector oligo (DO) probe mix (v1.1; 2,977 probes), for 10 min at 70°C followed by a temperature gradient with a ramp rate of 0.5°C/min to 45°C over 50 min followed by a nuclease digestion to remove excess, unbound, or incorrectly bound DOs enzymatically at 37°C for 90 min. The DO pairs bound to adjacent target sequences were then ligated (60 min at 37°C, followed by a 15 min enzyme denaturation at 80°C) to generate a pool of amplification templates. Each amplification template (10 μl of ligated DOs) was transferred to its respective well of the 96-well PCR plate containing PCR Pre-Mix and Primers. Amplification was conducted using a CFX96 Real-Time PCR Detection System (Bio-Rad, Mississauga, ON, Canada) to add a sequence tag unique to each sample and the sequencing adaptors using the following PCR program settings: 37°C for 10 min, 95°C for 2 min; 6 cycles of 95°C for 30 sec, 54°C for 30 sec, 72°C for 120 sec; 16 cycles of 95°C for 30 sec; 72°C for 2 min; 72°C for 1 min. All 288 TempO-Seq^®^ libraries prepared from the three 96-well plates were pooled (5 μl of each sample) and purified using the Macherey-Nagel NucleoSpin^®^ Gel and PCR Clean-Up kit (Clontech Laboratories Inc., Bethlehem, PA, USA), according to the manufacturer's directions for PCR clean-up with three modifications outlined in the TempO-Seq^®^ Assay User Guide. The pooled, purified TempO-Seq^®^ libraries were sequenced on two NextSeq^®^ 500/550 High Output flow cells (v2 kits, 75 cycles) using an Illumina NextSeq^®^ 500 Sequencing platform (Illumina, San Diego, CA, USA).

### Sequencing Data Preprocessing, Alignment, and Quality Control

Sequencing data have been deposited in the National Center for Biotechnology Information (NCBI) Gene Expression Omnibus (GEO) database under accession number GSE171360. Raw sequencing data were demultiplexed (i.e., assigned to respective sample files) with blc2fastq v2.20.0.422, and trimmed for quality control using fastp (v0.20.0). The resulting FASTQ files were aligned to reference sequences for the TempO-Seq^®^ Human Tox+Surrogate Panel (2,977 probes) provided by BioSpyder using their purpose-built analysis pipeline (TempO-SeqR, v3.0) to generate a table of counts per gene per sample. Briefly, this pipeline used STAR v2.7.8a to perform alignment of raw reads to the reference sequences, and the qCount function of the QuasR package (v1.30.0) to produce a gene X sample count matrix of raw counts from the BAM files output by STAR.

Study-wide quality control was performed on the count matrix using several methods to measure consistency and remove low-quality samples, using Harrill et al. (64) as a guideline. Samples that clustered as singletons at a dissimilarity of 0.1 using 1-Spearman correlation using complete linkage were removed from the study. As described by Harrill et al. (64), we used a cutoff of uniquely mapped reads as 10% of the number of target sequences (i.e., 100,000 reads to pass filter, because the target is 1,000,000 for TempO-Seq^®^ experiments). We removed any samples outside of Tukey's Outer Fence (3X interquartile range) for: (1) the number of probes capturing the top 80% of the signal; (2) the Gini coefficient (which measures inequality in distributions); and (3) the number of active probes (those with at least 5 mapped reads). Based on these metrics, a single experimental sample was removed (one replicate of EUG C4).

The code used to perform processing of high-throughput sequencing data is available at https://github.com/mattjmeier/2021_Buick_et_al_HepaRG_CometChip_TGx-DDI.

### Statistical and Bioinformatic Analyses for TGx-DDI Classification

Read counts were normalized using DESeq2 (v1.30.1) (65) using the counts() function in R (66) to account for sequence-to-sequence variability in read depth between the samples. Samples with sub-optimal sequencing depth (total number of reads <500 K) or that were overtly cytotoxic (>60% cytotoxic; <40% viable cells) were excluded from the analysis. Data visualization using boxplots and hierarchical cluster analysis were conducted to identify samples with poor data quality. This resulted in the exclusion of one sample (a replicate of EUG C4) from the TGx-DDI classification analysis due to sub-optimal sequencing depth. One replicate of CISP C2 was also excluded from the analysis, as it was identified as “a point of high leverage” outlier (Supplementary Figure 1). Statistical modeling and bioinformatics tools were used to classify chemicals as DDI or non-DDI using the TGx-DDI genomic biomarker. Detailed information about the analyses can be found in Yauk et al. (67) and Buick et al. (68). Gene Symbols that had multiple probes for TGx-DDI biomarker genes were averaged. Hierarchical clustering was completed using the hclust() function in R (www.r-project.org). Agglomerative clustering was based on average linkage with Euclidean distances (69). Classifications (DDI vs. non-DDI) were achieved using the Nearest Shrunken Centroids (NSC) method (70) in the pamr function of R (www.bioconductor.org), as has been described previously (36, 67, 68). Briefly, the standardized centroid (SC) was calculated by applying the NSC method for DDI and non-DDI test chemicals in the training set and is the mean expression level for each gene in a class divided by its within-class standard deviation. For each DDI and non-DDI test article, the SC is shrunken in the direction of the overall centroid to create the NSC. Treated and control samples were then classified by comparing their gene expression profile to the class of NSCs and then assigned to a class closest to it in squared distance so that the probability of class membership was >0.90 (35, 36).

Three separate analyses were conducted to classify the compounds using the TGx-DDI biomarker, including NSC probability analysis (PA; visualized using heatmaps), principal component analysis (PCA), and hierarchical clustering (HC), as outlined in Yauk et al. (67) and Buick et al. (68). PCA was completed using the prcomp() function in R (71), where the training set data (35) was used to estimate the principal components (PC). These PC loadings were applied to the data generated with the 12 test compounds. A scatterplot generated using data from the TGx-DDI training set and test chemicals was generated to visualize the results. Classification was completed as follows: if a chemical resulted in a positive call in any one of three classification analyses (NSC PA heatmaps, PCA, or HC), it was classified as DDI; whereas, a chemical was classified as non-DDI if it did not lead to a positive result in any of the aforementioned analyses (54, 67, 68).

### Benchmark Concentration Modeling of CometChip® Data

Benchmark concentration analysis of CometChip^®^ data (BMC_CC_) was conducted using BMDExpress v2.3 (https://github.com/auerbachs/BMDExpress-2/releases) following BMD technical guidance (72, 73). Test chemicals with statistically significant increases in median % tail DNA were included for BMC_CC_ modeling with the exception of overtly cytotoxic concentrations, which resulted in the highest concentration (C5) of ENU being excluded from the BMC_CC_ analysis (all concentrations of EUG and PG were excluded from this analysis due to a lack of positive response). Concentration-response data were fit to a model that best described the data using the following models: Linear, Exponential (2, 4, and 5), 2° Polynomial, and the restricted Power (power restricted to ≥1). The benchmark response (BMR) was set to one standard deviation (1SD) (74). The BMCU_CC_ and BMCL_CC_ values signify the upper and lower 95% confidence limits of the BMC_CC_, respectively. The “width” of the confidence interval is the distance between the BMCU_CC_ and BMCL_CC_, and therefore defines the BMC_CC_ estimate's precision.

### Benchmark Concentration Modeling of TGx-DDI Biomarker Genes

For the TGx-DDI biomarker genes, normalized read counts were shifted by 0.5 and then log2 transformed using the counts() function in the DESeq2 package (65). BMC analysis of TGx-DDI biomarker genes (BMC_TGx_) was also conducted using BMDExpress v2.3, in accordance with recommendations outlined in the US National Toxicology Program (NTP) Research Report on National Toxicology Program Approach to Genomic Dose-Response Modeling (72, 73). Test chemicals with positive TGx-DDI classifications were included for BMC_TGx_ modeling with cytotoxic concentrations eliminated from the analysis [i.e., the highest concentration (C5) of ENU, EUG, and PG were excluded]. Biomarker genes were analyzed and filtered using the Williams trend test retaining features with a permutation *p*-value < 0.05 (with 250 permutations) with fold changes >1.5. To derive BMC_TGx_ values, TGx-DDI biomarker genes that passed the pre-filters were fit to the following models: Linear, Exponential (2, 4, and 5), 2° Polynomial, and the restricted Power (power restricted to ≥1). A best fit model was selected with the lowest Akaike Information Criterion (AIC) value (lowest complexity). To be consistent with the BMC_CC_ analysis, the BMR was set to 1SD for BMC_TGx_ analysis (74). BMCs were filtered based on the goodness of fit (*p*-value > 0.1), a BMC/BMCL ratio <20, a BMCU/BMCL ratio <40, the BMC < the highest concentration, and the BMC could not be < two orders of magnitude lower than the lowest concentration to avoid model extrapolation. A secondary analysis was also conducted in order to generate confidence intervals for the BMC_TGx_ values using the bootstrap method. For each gene, 100 bootstrap samples were generated assuming a normal distribution for each concentration group, where the mean and standard deviation were based on the sample estimates. These data were then imported into BMDExpress v2.3 with the same filtering criteria and model selection as in the BMC_TGx_ analysis. As bootstrap samples are independent, the BMDExpress results were then bootstrapped 2,000 times, where each gene in the biomarker has a probability for inclusion into a bootstrap sample based on the relative frequency of that gene estimated as the total number of BMCs for that gene that passed all the filtering criteria divided by 100. For each bootstrap sample, the median was estimated. From the resulting bootstrap distribution, 95% percentile confidence intervals were obtained for the median BMC_TGx_ (bootstrap).

## Results

Human HepaRG™ cells were exposed to increasing concentrations of 12 well-characterized compounds. Exposed cells were analyzed using CometChip^®^ to assess DNA damage and TempO-Seq^®^ for TGx-DDI classification purposes. BMC analyses were conducted for both tests to compare the potency for each test chemical for the different assays.

### Identification of Relevant Concentration Ranges for Genotoxicity Testing

**Figure 1:**
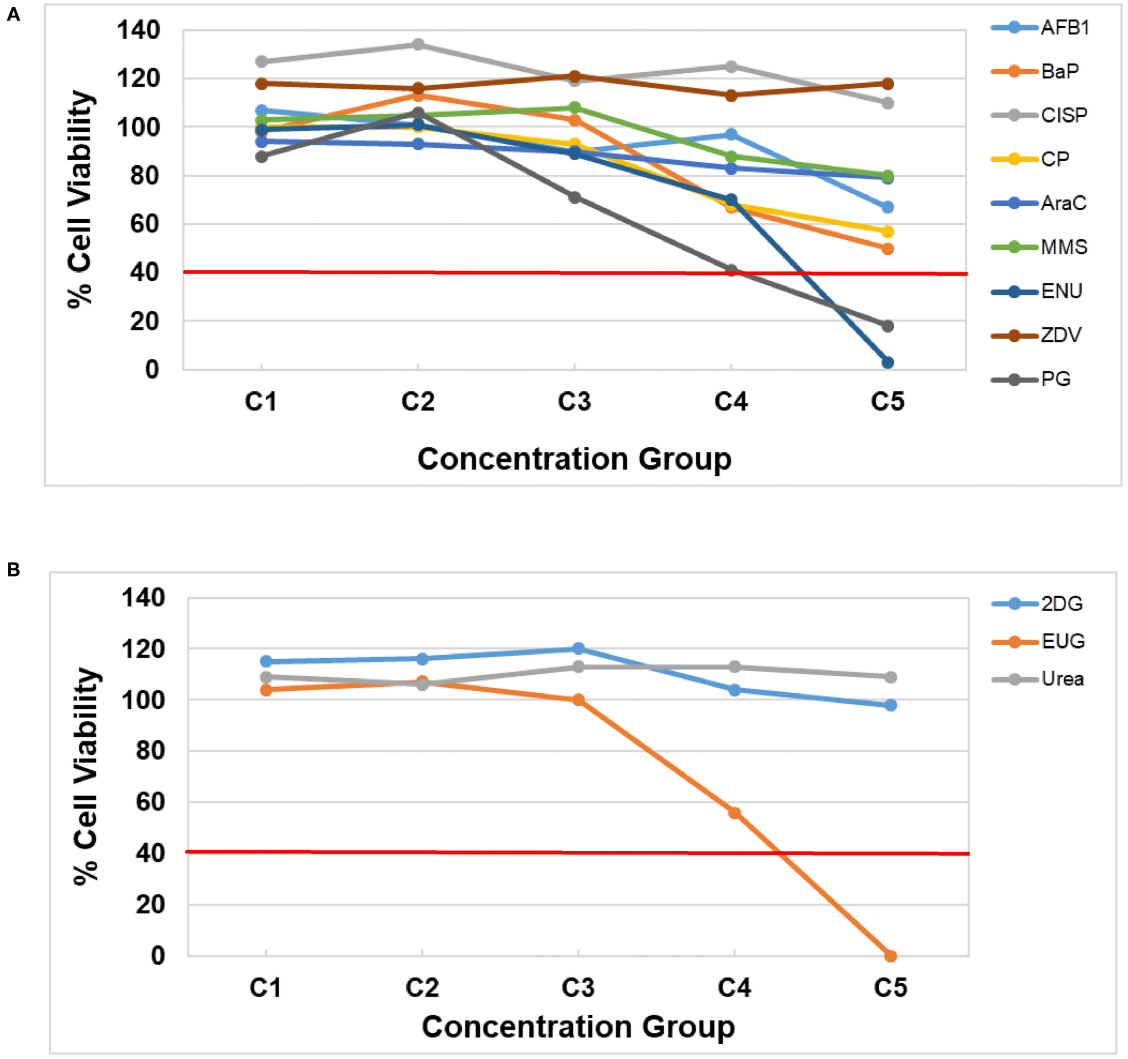
HepaRG™ cell viability measured using the CellTiter-Glo^®^ Luminescent Cell Viability Assay for **(A)** DDI (Group 1) chemicals and **(B)** non-DDI (Group 2 and 3) chemicals. DDI chemical abbreviations: aflatoxin B1 (AFB1), benzo[a]pyrene (BaP), cisplatin (CISP), cyclophosphamide (CP), cytosine arabinoside (AraC), methyl methanesulfonate (MMS), N-ethyl-N-nitrosourea (ENU), zidovudine (ZVD), and propyl gallate (PG; included with Group 1 chemicals). Non-DDI chemical abbreviations: 2-deoxy-D-glucose (2DG), eugenol (EUG), and Urea. The test concentrations for each chemical are shown in Table 2 (C1 is the lowest concentration and C5 is the highest concentration). The red line represents the cytotoxicity threshold of <40% cell viability.

Prior to assessing DNA damage and DNA damage-induced genes, it is first necessary to identify concentration ranges that include two or more non-cytotoxic concentrations. Cell viability was assessed using the CellTiter-Glo^®^ Luminescent assay following a 3-day repeat exposure to five concentrations of each test chemical by quantifying the luminescent signal from ATP, an indicator of metabolically active cells, in the treated HepaRG™ cells (Figure 1, Supplementary Table 1). All of the DDI (Group 1) compounds except ZDV and CISP caused declines in viability (Figure 1A). AFB1, BaP, CP, AraC, and MMS resulted in reduced cell viability, but none of the test concentrations exceeded the cytotoxicity threshold of >60% (equivalent to <40% cell viability). The highest concentration (C5) of ENU and PG resulted in <20% cell viability following treatment (Figure 1). 2DG (Group 2) and Urea (Group 3) did not cause any notable cytotoxicity at any of the concentrations tested. Finally, EUG (Group 3) caused declines in viability with the top concentration being eliminated due to overt cytotoxicity (<20% cell viability). All concentrations of test chemicals were used for CometChip^®^ analysis, including those causing overt cytotoxicity, for simplicity; however, any chemical treatment exceeding the cytotoxicity limits were excluded from the TempO-Seq^®^ gene expression analysis (i.e., C5 for ENU, EUG, and PG) due to the higher cost of this assay. Cytotoxic concentrations (<40% viability) were also eliminated for hazard calling and BMC modeling of both endpoints (i.e., C5 for ENU, PG, and EUG were not included; only positive CometChip^®^ and TGx-DDI responses were modeled).

### High-Throughput DNA Damage Quantification

**Figure 2:**
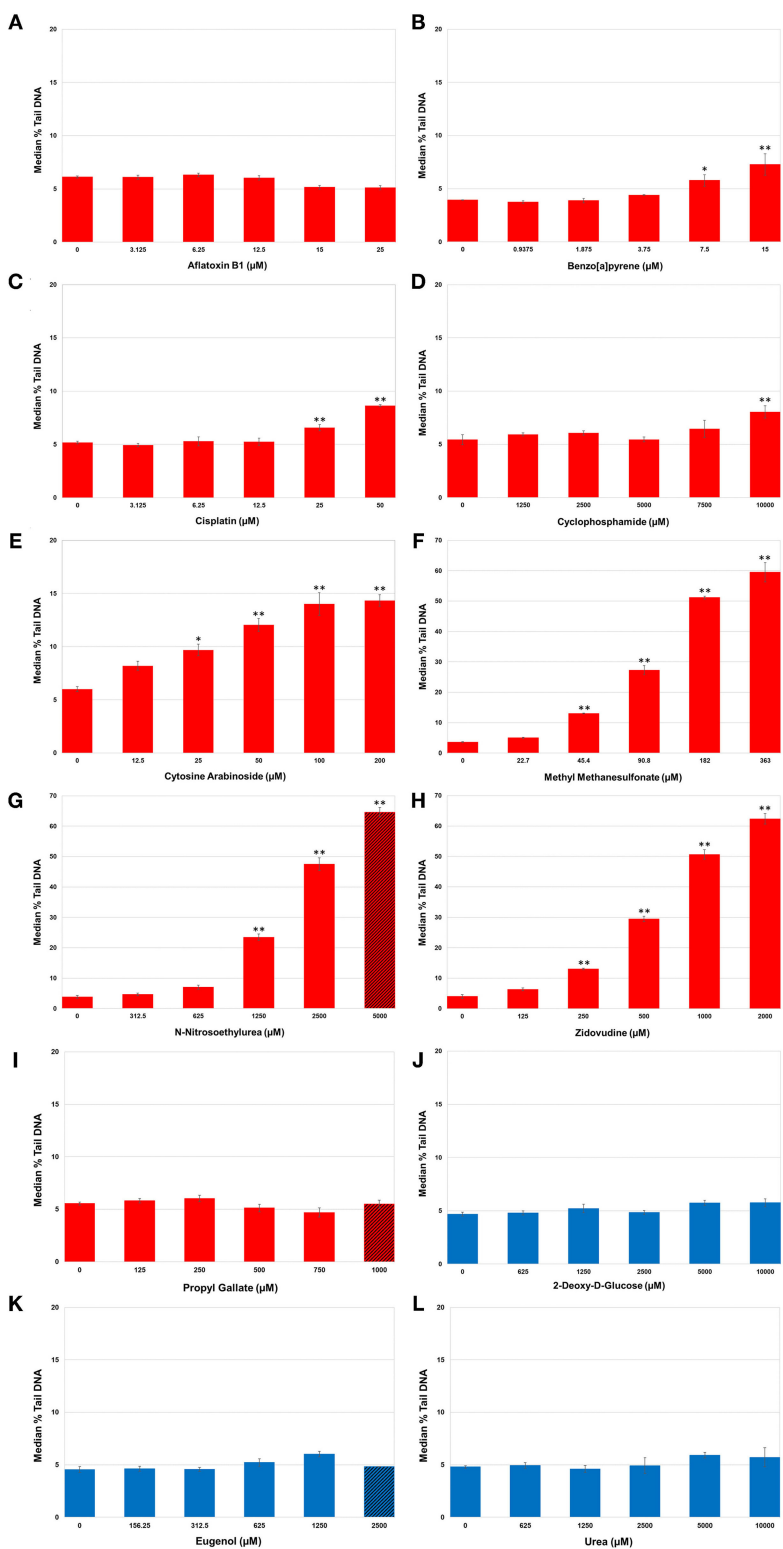
DNA damage in human HepaRG™ cells measured using the alkaline CometChip^®^ assay. Cells were exposed to increasing concentrations of DNA damage-inducing **(DDI; A–I)** and non-DDI test chemicals **(J–L)**. Median % tail DNA is shown 4 h following the last exposure. The data are expressed as the median % tail DNA ± SE (*n* = 4). Red bars represent Group 1 DDI chemicals, blue bars denote the Group 2 non-DDI chemical and the Group 3 potentially misleading positive chemicals. Diagonal lines indicate overtly cytotoxic concentrations. Note the difference in the scale of the y-axis for MMS, ENU, and ZDV due to the large magnitude of the response for these three DDI (Group 1) chemicals. *P < 0.05, **P < 0.001 compared to matched vehicle control.

Levels of DNA SSBs were quantified in human HepaRG™ cells following repeat exposures to the 12 test chemicals using the alkaline CometChip^®^ assay (Figure 2). Chemicals were considered positive if there was an increase in median % tail DNA that was statistically significant compared to matched vehicle control (*p* < 0.05). DNA damage, measured by median % tail DNA, was observed for seven of the nine DDI (Group 1) chemicals to varying degrees, with at least one concentration resulting in significant DNA damage compared to matched vehicle controls. MMS, ENU, and ZDV exposure caused the greatest accumulation of significant DNA damage in HepaRG™ cells (*p* < 0.001; Figures 2F–H, respectively; note the y-axis scale for these three compounds is greater than the other chemicals in this figure); however, the top concentration of ENU (C5) surpassed the cytotoxicity threshold (<20% cell viability). Almost all of the remaining DDI chemicals also induced significant increases in SSBs as detected by CometChip^®^, but to a lesser extent. AraC caused significant DNA damage at the top four concentrations tested (Figure 2E). BaP and CISP caused significant increases in % tail DNA at C4 and C5 (Figures 2B,C). CP exposure only caused significant % tail DNA increases at the highest concentration (*p* < 0.05; Figure 2D). AFB1 exposure did not cause measurable increases in DNA SSBs using the alkaline CometChip^®^ assay (Figure 2A), which is consistent with the relatively low magnitude of % tail DNA for agents that induce bulky lesions [this includes AFB1, BaP, CISP, and CP; (55, 75, 76)]. PG exposure also did not yield a statistically significant increase in DNA damage, which is consistent with the fact that it does not directly interact with DNA to create physical damage [Figure 2I; (77, 78)].

There was no accumulation of SSBs observed at any of the five concentrations tested for 2DG, the non-DDI chemical (Group 2: non-genotoxic), nor for EUG and Urea (Group 3: misleading positives); median % tail DNA was not statistically increased compared to their matched vehicle controls (Figures 2J–L).

In summary, significant DNA damage was detected following exposure to seven out of nine DDI compounds (Group 1). Accumulation of DNA damage was concentration-dependent for the majority of DDI compounds, with very large increases in % tail DNA for ENU, MMS, and ZDV. Exposure to non-DDI (Group 2) and misleading positive compounds (Group 3) did not cause increases in DNA SSBs in HepaRG™ cells even in the presence of overt cytotoxicity.

### TempO-Seq® Analysis for TGx-DDI Biomarker Classification

TempO-Seq^®^ S1500+ sequencing was conducted for the purposes of classifying the test chemicals as DDI or non-DDI using the TGx-DDI genomic biomarker. None of the negative assay controls (1X TempO-Seq^®^ Lysis buffer, no lysates) exceeded 1,200 mapped read counts and the positive assay controls (Human Reference Total RNA and Human Brain Total RNA) showed Pearson correlation coefficients between the replicates that were >0.98 for all pairwise comparisons. The outlier analysis (described above) resulted in the removal of two samples (one replicate each of CISP C2 and EUG C4). Thus, the final sample size was *n* = 4, except for CISP C2 and EUG C4 that had an *n* = 3.

**Figure 3:**
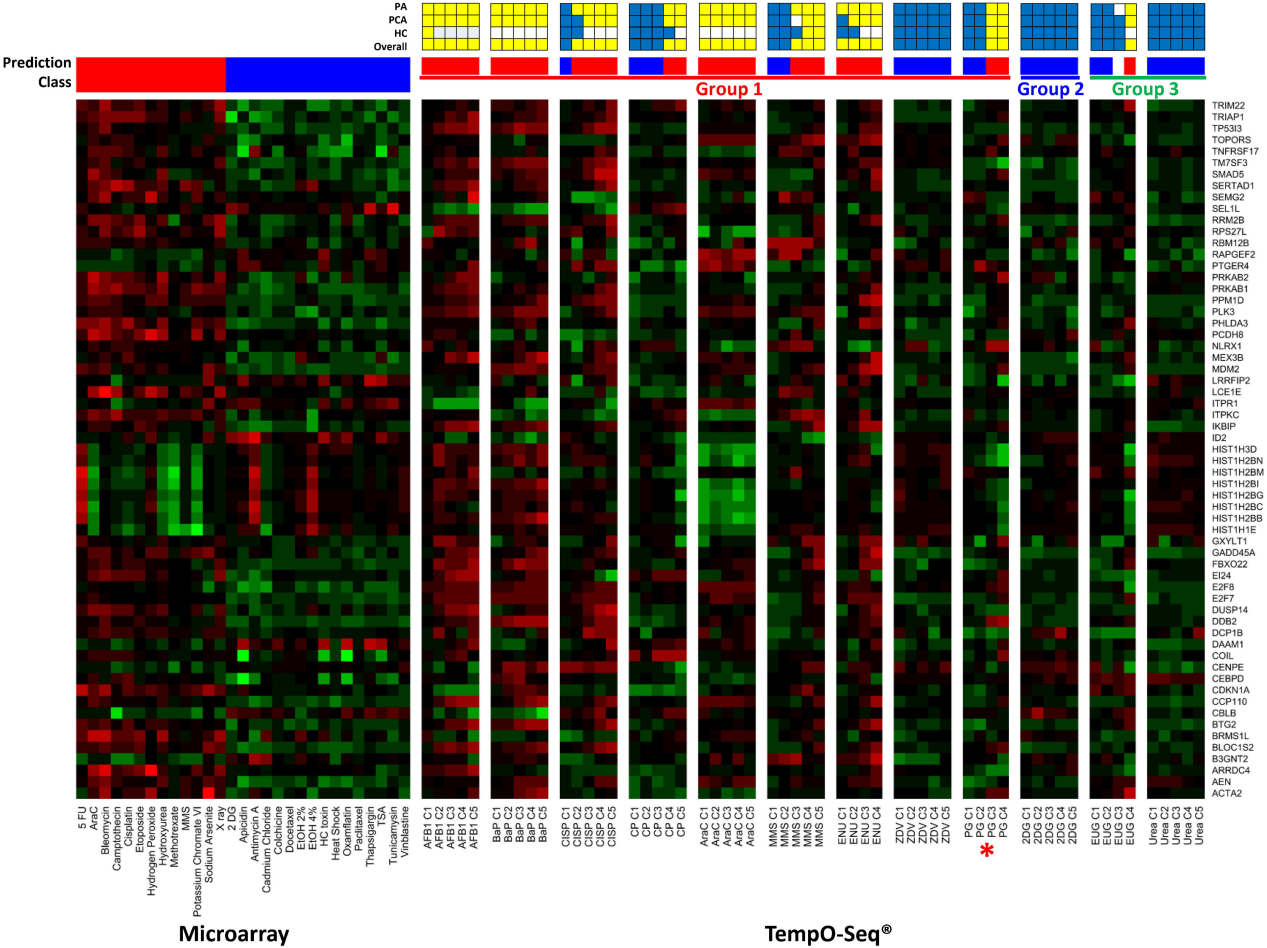
TGx-DDI classification results are depicted using TempO-Seq^®^ gene expression technology for the 12 test chemicals. The heatmap on the left shows the gene expression responses of the TGx-DDI biomarker genes for the 28 reference compounds used to generate the biomarker using DNA microarray analysis in TK6 cells. The test chemicals assessed using TempO-Seq^®^ gene expression technology in human HepaRG™ cells are shown in the subsequent heatmaps (columns). Gene Symbols corresponding to the GenBank accession numbers for the TGx-DDI biomarker genes are shown on the right. The color scale specifies fold changes relative to control: up-regulated genes are red, down-regulated genes are green, and genes exhibiting no changes relative to controls are black. Overall calls for all treatment conditions are shown using red (DDI) and blue (non-DDI) bars above each heatmap. Chemical groupings from Kirkland et al. (57, 58) include Group 1 DDI chemicals (red), a Group 2 non-DDI chemical (blue), and Group 3 potentially misleading positive chemicals (green). DDI chemical abbreviations: aflatoxin B1 (AFB1), benzo[a]pyrene (BaP), cisplatin (CISP), cyclophosphamide (CP), cytosine arabinoside (AraC), methyl methanesulfonate (MMS), N-ethyl-N-nitrosourea (ENU), zidovudine (ZVD), and propyl gallate (PG; *included with Group 1 chemicals). Non-DDI chemical abbreviations: 2-deoxy-D-glucose (2DG), eugenol (EUG), and Urea. The grids above the heatmaps indicate the results of the three different TGx-DDI analyses: Probability Analysis (PA, based on nearest shrunken centroid analysis), Principal Component Analysis (PCA) and Hierarchical Clustering (HC). The overall call is DDI if any one of these analyses yields a DDI call. Yellow boxes indicate a positive DDI classification, blue denotes a negative non-DDI classification and white signifies an unclassified response (i.e., does not yield a DDI or non-DDI call).

Three independent analyses, including NSC PA, PCA, and HC were considered in the overall TGx-DDI classification. Figure 3 is a heatmap that represents the TGx-DDI predictions for all 12 test chemicals using NSC PA. Supplementary Figures 2A–L depicts the PCA results (panel i) and the HC results (panel ii). If a test chemical had a positive call in one or more analyses, it was predicted to be DDI; whereas, a chemical that had a negative call in all three analyses was classified as non-DDI. The TGx-DDI biomarker accurately classified eight out of nine DDI chemicals. Four of the nine DDI compounds, AFB1, BaP, AraC, and ENU, were classified as DDI at all five concentrations tested. CISP classified as DDI at the top four concentrations; MMS classified as DDI at the three highest concentrations; and CP and PG were both predicted to be DDI at top two concentrations tested (C5 was overtly cytotoxic so gene expression analysis was not conducted for the highest concentration of PG). ZVD was the only DDI compound that misclassified as non-DDI at all concentrations tested.

The TGx-DDI biomarker correctly classified 2DG, a non-DDI (Group 2) chemical, as non-DDI at all concentrations tested (Figure 3). For Group 3 chemicals (EUG and Urea), which are potentially misleading positives according to Kirkland et al. (57, 58), only urea classified as non-DDI at all concentrations tested (Figure 3). EUG classified as non-DDI at the two lowest concentrations, was unclassified at C3, but rendered a positive DDI classification at C4, the highest concentration tested (C5 was overtly cytotoxic, thus, gene expression analysis was not conducted for the highest concentration).

An overview of the CometChip^®^ and TGx-DDI outcomes is shown in Table 3. Overall, measurements of DNA damage analyzed by CometChip^®^ were concordant with TGx-DDI classification results for eight test chemicals. Four test chemicals rendered discordant results: AFB1, PG, and EUG yielded negative CometChip^®^ results but positive TGx-DDI calls; and ZDV was positive by CometChip^®^ but negative with TGx-DDI. By combining the CometChip^®^ assay and the TGx-DDI biomarker (i.e., positive in one assay = positive; negative in both = negative), 11 of the 12 test chemicals were accurately classified.

**Table 3:** Summary of CometChip^®^ analysis and TempO-Seq^®^ TGx-DDI classification results for 12 test chemicals.

	**CometChip** ^**®**^ **(DNA damage)**					**TGx-DDI classification (gene expression)**				
**Group 1 chemicals**	**C1**	**C2**	**C3**	**C4**	**C5**	**C1**	**C2**	**C3**	**C4**	**C5**
Aflatoxin B1	-	-	-	-	-	+	+	+	+	+
Benzo[a]pyrene	-	-	-	+	+	+	+	+	+	+
Cisplatin	-	-	-	+	+	-	+	+	+	+
Cyclophosphamide	-	-	-	-	+	-	-	-	+	+
Cytosine arabinoside	-	+	+	+	+	+	+	+	+	+
Methyl methanesulfonate	-	+	+	+	+	-	-	+	+	+
N-Ethyl-N-nitrosourea	-	-	+	+	X	+	+	+	+	X
Zidovudine (azidothymidine)	-	+	+	+	+	-	-	-	-	-
Propyl gallate	-	-	-	-	X	-	-	+	+	X
**Group 2 chemicals**	**C1**	**C2**	**C3**	**C4**	**C5**	**C1**	**C2**	**C3**	**C4**	**C5**
2-Deoxy-D-glucose	-	-	-	-	-	-	-	-	-	-
**Group 3 chemicals**	**C1**	**C2**	**C3**	**C4**	**C5**	**C1**	**C2**	**C3**	**C4**	**C5**
Eugenol	-	-	-	-	X	-	-	U	+	X
Urea	-	-	-	-	-	-	-	-	-	-

*C1 (lowest concentration tested) to C5 (highest concentration tested); X = overtly cytotoxic (<40% cell viability). For the CometChip^®^ data, + indicates statistically significant increase in DNA damage (p < 0.05), as measured using the median % tail DNA and - represents no significant DNA damage. For the gene expression data, + represents a DDI classification, - represents a non-DDI classification, and U indicates a sample that was unclassified using the TGx-DDI genomic biomarker (TempO-Seq^®^ data)*.

### BMC Analysis of CometChip® Data and TGx-DDI Biomarker Genes

BMC analysis is used to mathematically model the concentration-response curves to determine the concentration at which a predefined increase above controls occurs for potency ranking purposes. BMC modeling was conducted to derive BMCs for both apical and transcriptional endpoints and were denoted as follows: BMC CometChip^®^ (BMC_CC_) and BMC TGx-DDI biomarker genes (BMC_TGx_). The BMR used was 1SD for both BMC_CC_ and BMC_TGx_. BMCL and BMCU were also calculated, and these are referenced in the same manner (i.e., BMCL_CC_ and BMCU_CC_; BMCL_TGx_ and BMCU_TGx_, respectively). Two strategies were used to calculate transcriptomic BMCs (i.e., the NTP's approach to genomic dose-response modeling and a bootstrap method); note that only the bootstrap method allowed for the calculation of the 95% confidence intervals for the TGx-DDI gene set (CIs; i.e., distance between the BMCL_TGx_ and BMCU_TGx_). Comparison of calculated BMC values and confidence limits for CometChip^®^ and the TGx-DDI biomarker genes (both methods) are shown in Table 4, in addition to the number of TGx-DDI biomarker genes that could be modeled and the ratio of BMC_TGx_/BMC_CC_ for comparison of median % tail DNA and transcriptomic BMC values.

**Table 4:** Comparison of benchmark concentrations for CometChip^®^ (BMC_CC_) and TGx-DDI biomarker genes (BMC_TGx_).

	**CometChip^®^ BMC**	**TGx-DDI median BMC**		**TGx-DDI bootstrap median BMC**		
	**Median BMC_**CC**_ (BMCL_**CC**_ - BMCU_**CC**_)**	**#TGx-DDI genes modeled**	**Median BMC_**TGx**_**	**#TGx-DDI genes modeled**	**Median BMC_**TGx**_ (BMCL_**TGx**_ - BMCU_**TGx**_)**	**Ratio BMC_**TGx**_/BMC_**CC**_**
**Group 1**						
Aflatoxin B1	n.m.	26	2.9	53	2.7 (1.7–5.3)	**-**
Benzo[a]pyrene	1.7 (1.4–2.3)	15	0.56	50	0.68 (0.36–1.5)	0.39
Cisplatin	8.5 (6.8–11.2)	19	4.5	54	5.4 (2.5–11.5)	0.63
Cyclophosphamide	8,079 (6,093–9,670)	10	4,808	52	4,949 (2,086–8,408)	0.61
Cytosine arabinoside	12.1 (9.0–17.9)	9	16.6	47	12.9 (3.4–36.2)	1.07
Methyl methanesulfonate	21.3 (16.1–28.8)	22	67.1	49	76.5 (48.2–115)	3.56
N-Ethyl-N-nitrosourea	536 (389–761)	21	285	50	271 (163–494)	0.50
Zidovudine (azidothymidine)	82.5 (64.0–110)	n.m.	n.m.	n.m.	n.m.	**-**
Propyl gallate	n.m.	20	273	56	336 (246–443)	**-**
**Group 2**						
2-Deoxy-D-glucose	n.m.	n.m.	n.m.	n.m.	n.m.	**-**
**Group 3**						
Eugenol	n.m.	17	535	56	529 (382–748)	**-**
Urea	n.m.	n.m.	n.m.	n.m.	n.m.	**-**

*Chemicals that displayed a statistically significant increase in median % tail DNA were modeled for CometChip^®^ BMC (BMC_CC_) analysis. Chemicals that resulted in a positive DDI classification using the TGx-DDI biomarker were modeled for TGx-DDI BMC (BMC_TGx_) analysis. Chemicals that did not fit the criteria to be modeled for BMC_CC_ or BMC_TGx_ were not modeled (n.m.). TGx-DDI Median BMCs were calculated using the criteria and strategy outlined in the National Toxicology Program's Approach to Genomic Dose-Response Modeling (72). TGx-DDI Bootstrap Median BMCs were calculated using a bootstrap method to allow lower and upper confidence intervals to be calculated*.

Of the seven DDI compounds that could be modeled for the CometChip® data, BaP, CISP, AraC, and MMS were the most potent genotoxicants in HepaRG™, with BMC_CC_ of 1.7, 8.5, 12.1, and 21.3 μm, respectively. BMC_CC_'s for ZDV and ENU were 82.5 and 536 μm, respectively; whereas, the data suggest that CP was the least potent DDI compound in HepaRG™ cells with a BMC_CC_ of 8079 μm. Potency ranking for SSBs was thus BaP > CISP > AraC > MMS > ZDV > ENU > CP > (negative in CometChip^®^ – AFB1, PG, 2DG, EUG, Urea).

BMC_TGx_ analysis for DDI test chemicals with a positive TGx-DDI classification was conducted using two different methods. Both the median BMC_TGx_ and the bootstrap median BMC_TGx_ resulted in very similar potency rankings of the DDI chemicals (Table 4). Of the eight DDI compounds that could be modeled for the TGx-DDI data, BaP, AFB1, CISP, AraC, and MMS were the most potent genotoxicants in HepaRG™, with BMC_TGx_ (bootstrap median) of 0.68, 2.7, 5.4, 12.9, and 76.5 μm, respectively. BMC_TGx_ for ENU and PG were 271 and 336 μm, respectively. CP was the least potent DDI compound with a BMC_TGx_ of 4949 μm. For the median BMC_TGx_, the potency ranking was as follows: BaP > AFB1 > CISP > AraC > MMS > PG > ENU > EUG > CP > (TGx-DDI negative – ZDV, 2DG, Urea); whereas, the ranking of ENU and PG were reversed for bootstrap median BMC_TGx_ (i.e., BaP > AFB1 > CISP > AraC > MMS > ENU > PG > EUG > CP; Table 4). The number of TGx-DDI biomarker genes (64 in total) that fit models ranged from 9 (AraC) to 26 (AFB1) using the median BMC approach, but increased substantially from 47 (AraC) to 56 (PG and EUG) using the bootstrap median BMC approach (Table 4).

**Figure 4:**
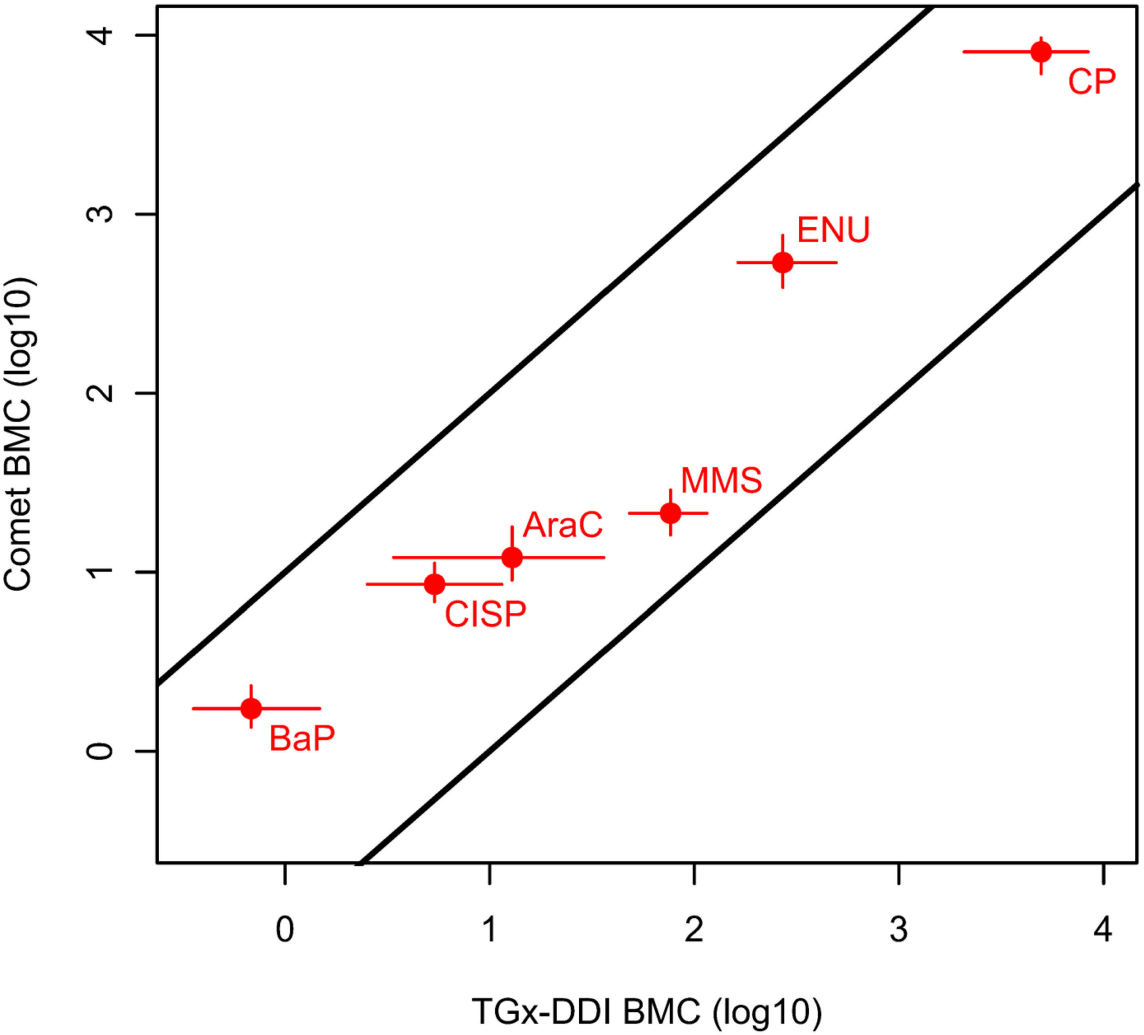
Relationship between the BMCs of the CometChip^®^ data (BMC_CC_) and the TGx-DDI biomarker genes (BMC_TGx_) for the test chemicals that yielded positive hazard calls in both tests and could be modeled. Specifically, the BMC_CC_ with a BMR of 1SD with two-sided 95% CIs are shown for the CometChip^®^ vs. median bootstrap BMC_TGX_ (BMR of 1SD) with two-sided 95% CIs for the TGx-DDI biomarker classification endpoint. The BMCs for the agents that were classified as DDI from both approaches were within 10-fold. The two parallel black lines with intercepts of 1 and −1 on the double log10 scale represent a 10-fold deviation from the 1:1 line.

We then directly compared chemicals that were positive in both assays and could be fit to BMC models (i.e., 6 of the 12 chemicals). Remarkably, when comparing chemicals that could be modeled for both CometChip® and transcriptomic endpoints (bootstrap method), the chemical ranking was identical and the ratios of BMC_TGx_/BMC_CC_ were within 4-fold, ranging from 0.4 to 3.6 (Table 4 and Figure 4). BaP, CISP, CP, and ENU had marginally lower BMC_TGx_ than BMC_CC_; whereas, the BMC_TGx_ and BMC_CC_ were virtually the same for AraC. MMS was the only test chemical with a lower BMC_CC_ than BMC_TGx_, and this is consistent with the fact that MMS is the only chemical that is primarily repaired by the base excision repair (BER) pathway; see discussion (Table 4 and Figure 4). The confidence intervals on the BMC_TGx_ are larger than the BMC_CC_. Overall, the BMC_CC_ and the BMC_TGx_ are highly correlated and result in the same chemical rankings for both the CometChip^®^ assay and the transcriptomic TGx-DDI biomarker assay.

## Discussion

To address twenty-first century toxicology needs, an efficient and accurate genotoxicity testing paradigm is urgently required to assess the expanding backlog of data poor chemicals in need of genotoxic evaluation. A high-throughput, integrated test approach that pairs apical and mechanistic data in a human-relevant cell model with metabolic capabilities would be beneficial to address this requirement. TGx biomarkers are useful for this purpose as they enable rapid extraction of mechanistic data from HTTr data (36), which is more compelling when paired with a measure of DNA damage. To address this need, here we have combined a measure of DNA damage (the CometChip^®^ assay) with the TGx-DDI genomic biomarker in physiologically-relevant human HepaRG™ cell cultures for hazard identification and quantitative analysis of genotoxic potential of DDI and non-DDI chemicals.

We exposed differentiated human HepaRG™ cells using a 3-day daily repeat exposure protocol to 12 test chemicals with varying modes-of-action, including nine DDI (Group 1), one non-DDI (Group 2), and two potentially misleading positives (Group 3). The TGx-DDI transcriptomic biomarker was analyzed with the high-throughput TempO-Seq^®^ platform to establish the value of its integration with the high-throughput CometChip^®^ assay to assess DNA damage. Although each assay had merit on its own, integration of these genotoxicity tests correctly classified all of the DDI agents (Group 1), the non-DDI agent (Group 2), and identified one of two Group 3 chemicals (i.e., “misleading” positive) as non-DDI. BMC modeling of both endpoints revealed identical potency rankings for SSBs compared to transcriptional changes (i.e., BMC_TGx_/BMC_CC_ ratios were within 4-fold). We conclude that integration of the CometChip^®^ assay with the TGx-DDI genomic biomarker in HepaRG™ cells provides an effective and higher-throughput approach to genotoxicity testing to accurately identify and prioritize chemicals that cause DNA damage and to evaluate their potency. Below we discuss the concordant and discordant results in the context of the complementarity of these assays.

We first explored the concordance of hazard calls made using the CometChip^®^ assay with the TGx-DDI transcriptomic biomarker. Eight of 12 test chemicals yielded concordant hazard calls (i.e., caused SSBs and classified as DDI, or did not cause SSBs and classified as non-DDI). Of the nine DDI chemicals, six produced concordant results albeit at differing concentrations: BaP, CISP, CP, AraC, MMS, and ENU. Of these, the TGx-DDI biomarker measured using TempO-Seq^®^ was somewhat more sensitive at detecting DNA damage for five of the six chemicals. Specifically, it classified chemicals as DDI at lower concentrations than at which DNA damage was observed using the CometChip^®^ assay (MMS was the only exception). This is consistent with our BMC analysis, where MMS was the only chemical with a BMC_TGx_/BMC_CC_ ratio >1. This is interesting as MMS is the only test chemical included in this study where DNA damage is almost exclusively repaired by means of Base Excision Repair (BER) (79). BER enzymes eliminate damaged bases, which result in persistent SSBs as requisite DNA repair intermediates. The alkaline comet assay does not directly detect damaged base lesions, rather they are indirectly measured when the DNA repair enzymes create strand breaks in the repair process. This is in contrast to the highly coordinated Nucleotide Excision Repair (NER) pathway that is extremely efficient in repairing damage and therefore minimizes the detectable SSB repair intermediates (55). Thus, there was a large degree of concordance in genotoxicity hazard calls between the assays, with a marginally increased sensitivity of TGx-DDI at lower concentrations.

Conversely, there were three instances of discordant test results across the two assays for the nine DDI chemicals tested: AFB1, PG, and ZDV. AFB1 was negative by CometChip^®^ but had a very strong transcriptional DNA damage response and was classified as DDI using the TGx-DDI biomarker. A negative comet result is not unexpected for AFB1, as it is a genotoxic carcinogen that induces bulky DNA adducts. The alkaline comet assay is best suited for the identification of SSBs, abasic sites, and alkali-sensitive sites (31); it is not very sensitive in the detection of bulky lesions as they do not directly affect DNA migration. Thus, modifications to the standard Comet assay greatly increase sensitivity and therefore help to reliably detect bulky DNA adducts that are actively repaired by means of NER in a highly coordinated fashion (possibly with short-lived NER-induced SSB repair intermediates) (55, 80–83). Specifically, co-exposure to hydroxyurea and AraC traps NER intermediates, allowing for SSB repair intermediates to persist, which greatly improves the sensitivity of the assay for bulky lesions [e.g., (55)]. It is also possible that DNA damage may have been detectable with the CometChip^®^ assay following AFB1 exposure had we used a higher test concentration that reduced cell viability to the 40% target, as a modest increase was seen with other adduct-forming chemicals (e.g., BaP and CP) that did achieve this level of cytotoxicity. It is also important to consider the dynamics of repair and metabolism; it is possible that analysis at a different time point or in a different human cell line could yield different findings. Distinct biotransformation properties and genotoxic responses are associated with both cell line and time, which can influence the detection capabilities of the assay (75, 84). Nonetheless, we note that the TGx-DDI biomarker identified AFB1 as a strong positive, supporting the complementarity of these assays.

Conflicting responses were also observed for PG, an additive used to prevent oxidation (negative CometChip^®^ and positive TGx-DDI) (78, 85). PG is positive in the Ames assay with S9 metabolic activation (59) and induces MN and chromosomal aberrations *in vitro* and *vivo* (60, 86). Thus, the TGx-DDI biomarker correctly classified PG as DDI lending support to the removal of PG from Kirkland et al. (58) Group 3 chemical list. Some antioxidant chemicals, including PG, promote the generation of reactive oxygen species at elevated concentrations (85, 86). Thus, it is possible that we may have detected oxidative DNA damage following PG exposure using a formamidopyrimidine-DNA glycosylase (Fpg)-modified Comet assay, as this lesion-specific enzyme can convert undetectable base lesions caused by oxidative DNA damage into detectable SSBs (31).

Finally, ZDV also yielded discordant outcomes; in this case, a strong DNA damage response by CometChip^®^ was observed with a non-DDI prediction using the TGx-DDI biomarker. This result is plausible and not unexpected as ZDV, also known as azidothymidine, is an anti-HIV medication that belongs to a class of nucleoside analog reverse-transcriptase inhibitors (87), which can dampen the gene expression response (88, 89). Indeed, visual inspection of the heatmap of TGx-DDI genes reveals a broad decrease in transcript levels following ZDV exposure (Figure 3).

These discordant results highlight the fact that a single *in vitro* genotoxicity test is not likely to detect all DDI compounds due to the vast array of genotoxic MoAs and the limitations inherent to specific genotoxicity assays. However, when a standardized DNA damage test (i.e., the Comet assay) is paired with a transcriptomic biomarker for DNA damage (i.e., the TGx-DDI biomarker), this built-in test redundancy helps to ensure correct classification and indicates when further follow-up may be necessary to further assess certain chemicals. Indeed, Allemang et al. (90) compared classical and twenty-first century genotoxicity tools (*in vitro* MN, ToxTracker assay, and genomics-based methods including TGx-DDI) and found that no single test correctly classified all genotoxicants when used in isolation; however, the ability to identify genotoxicants improved dramatically when the *in vitro* MN assay was combined with another predictive test such as the TGx-DDI biomarker. They determined that a “fit for purpose” approach was required to combine the appropriate assays to maximize the predictive capacity of the tests for genotoxicity assessment.

The TGx-DDI transcriptomic biomarker was originally developed to distinguish DDI from non-DDI compounds to aid in the interpretation of positive *in vitro* genotoxicity outcomes. In our previous work, Li et al. (36) demonstrated that the TGx-DDI biomarker correctly identified nine out of 10 chemicals classified as having “irrelevant positive” *in vitro* chromosome damage results. In this study, there are two potentially misleading positive chemicals from the Group 3 list: EUG and Urea. Group 3 chemicals *should* test negative, but have been reported to induce gene mutations, chromosomal aberrations, or MN, often at high concentrations or high levels of cytotoxicity (57, 58). Urea rendered negative results (i.e., no SSBs and non-DDI classifications) at all concentrations tested for both assays. However, this was not the case for EUG. EUG is a naturally occurring phenolic molecule found in plants (Table 1) (91, 92). While it did not cause any detectable SSBs using the CometChip^®^ assay herein, exposure to EUG resulted in a DDI classification at the highest non-cytotoxic concentration (C4) with TGx-DDI. Although EUG is generally negative for genotoxicity endpoints in p53-competent cells (86), it has tested positive in the mouse lymphoma assay and for chromosomal aberrations at high levels of cytotoxicity – it has been hypothesized that these levels of exposure may overwhelm detoxification leading to positive results (58, 93). The highest concentration of EUG analyzed for TGx-DDI was very close to the cytotoxicity threshold of 60% (56% for EUG C4); thus, it seems that high levels of cytotoxicity may be a plausible reason for the misclassification of EUG herein. Moreover, EUG was tested at a high concentration for C4 (1250 μm), which may have contributed to the misleading positive TGx-DDI classifications at this test concentration, as it may have depleted the detoxification potential of the cells, leaving EUG to cause primary DNA lesions in some cell types (91). It is possible that the discordant observations for EUG are a result of the different assay sensitivities. Alternatively, it is possible that EUG is exerting a genotoxic effect *via* a different MoA (i.e., a genotoxic mechanism that does not lead to SSBs), which results in a positive TGx-DDI classification in the absence of SSBs. Based on our results and those of the aforementioned studies, we speculate that the discordant results obtained for EUG are in fact relevant and thus require further analysis to explore the DDI potential of this chemical at high concentrations and/or levels of cytotoxicity.

The field of genetic toxicology is shifting toward more quantitative analyses of genetic toxicology data for potency assessments (94–97). Previous work has shown that transcriptional PODs are well-aligned with apical PODs (98–101). Moreover, Bemis et al. (102) demonstrated the correlation between *in vitro* and *in vivo* BMDs for flow cytometric micronucleus data and suggested that the clastogenic potential of a chemical can be calculated from animal studies or cell-based models of chromosome damage. Our previous work in human TK6 cells also demonstrated the concordance of BMC_MN_ and BMC_TGx_ (i.e., within 10-fold) following exposure to three chemicals (68). Herein, we applied BMC modeling to the 12 test chemicals to compare potencies using the CometChip^®^ assay vs. the TGx-DDI biomarker. The BMC_TGx_ values calculated were: (1) the TGx-DDI median BMC, and (2) the TGx-DDI bootstrap median BMC. The bootstrap median BMC method allowed us to model a much higher number of TGx-DDI biomarker genes (e.g., 9 to 26 genes modeled for median BMC method vs. 47 to 56 for the bootstrap median BMC method). It also allowed us to generate 95% confidence intervals for the BMC_TGx_ values (i.e., BMCL_TGx_ and BMCU_TGx_), which is particularly useful for comparing chemical potency rankings. However, given that the concentration ranges differed for the test chemicals in this study, the BMCs were primarily used to compare each chemical's response across the two assays (i.e., BMC_CC_ vs. BMC_TGx_ for each chemical) and BMC comparisons within each assay must be interpreted with caution. For chemicals that had a positive response in both assays, we observed a good correlation between the BMC_CC_ and the BMC_TGx_ in that the ratio of BMC_TGx_ (bootstrap method)/BMC_CC_ was between 0.39 and 3.6 for BaP, CISP, CP, AraC, MMS, and ENU. Of the six chemicals that were modeled for both methodologies, the BMC_TGx_ was more sensitive for four of the chemicals (i.e., BaP, CISP, CP, ENU); the BMCs were virtually identical for AraC, and the BMC_CC_ was more sensitive for MMS. However, it should be noted that the lower BMC_TGx_ values were offset by larger confidence intervals on the TGx-DDI biomarker BMCs, which is expected as this is a composite biomarker that includes many gene BMCs. Nonetheless, chemical rankings are identical (i.e., the ranking from lowest to highest BMC) for the CometChip^®^ assay vs. the TGx-DDI biomarker using the bootstrap method (e.g., BaP > CISP > AraC > MMS > ENU > CP). This study provides further experimental evidence to support the use of BMCs as transcriptional points of departure since they are highly predictive of apical PODs.

In this study, the TGx-DDI assay was conducted using high-throughput targeted RNA-sequencing (TempO-Seq^®^) to improve the throughput, accuracy, and dynamic range of the gene expression analysis directly from HepaRG™ cell lysates, which also eliminates the requirement to extract RNA thereby improving the efficiency (103, 104). When HTTr is used in combination with a metabolically competent human cell line, such as HepaRG™ cells, it greatly decreases the time and cost required to assess a chemical (no additional test in the presence of S9 needed), while improving the human relevance of this NAM. Beyond the TGx-DDI classification, rich mechanistic data from the transcriptomic data are available for further mining. For example, standard pathway analyses can be applied to explore additional key events and other biomarkers can be analyzed in the same data sets [e.g., we have recently developed the TGx-HDACi transcriptomic biomarker; (105)]. One caveat is that chemicals that inhibit transcription are not amenable to analysis by transcriptomics, which can lead to misclassification (e.g., ZDV, a nucleoside transcriptase inhibitor, misclassified at all concentrations). However, our work and the work of others [e.g., (106, 107)] demonstrate that the use of transcriptomic biomarkers provides a rapid and non-subjective approach to the extraction of information about key toxicological events.

In summary, we demonstrate the potential of a new test paradigm that integrates the TGx-DDI biomarker with the high-throughput CometChip^®^ assay. We validate performance by HTTr profiling in the physiologically-relevant HepaRG™ cell model. Concentration-response modeling for the two tests established the concordance of BMCs for DNA SSBs measured using the CometChip^®^ assay and transcriptional changes in TGx-DDI biomarker genes. This is another step in accomplishing a more integrated genotoxicity testing strategy to derive mechanistic information to better inform human health risk assessment in a higher-throughput manner.

## Data Availability Statement

The data presented in this study are deposited in the NCBI Gene Expression Omnibus under accession number GSE171360. Data are available for download at: https://www.ncbi.nlm.nih.gov/geo/query/acc.cgi?acc=GSE171360.

## Author Contributions

CY, JB, AW, MM, CS, LR, SF, and BE were involved in project conception, in the development of the analytical approach, and in the data interpretation. CY, JB, and CS designed the study, in consultation with the other authors. CY obtained funding for the project. LR and CS conducted the HepaRG™ exposures, cell viability and CometChip^®^ assays. JB conducted the TempO-Seq^®^ gene expression experiments and prepared the manuscript with important intellectual input from CY, LR, CS, SF, and BE. MM was responsible for the read alignment and bioinformatics analysis of the sequencing data. AW conducted the statistical analyses and prepared some of the figures. RG was instrumental in the establishment of the TempO-Seq^®^ methodology and analysis in the laboratory. JB, CY, AW, MM, RG, and JB had complete access to the study data. All authors read, reviewed and approved the final manuscript.

## Conflict of Interest

LR and CS are employed at ILS, a contract research organization that conducts genetic toxicology testing services that include the use of HepaRG™ cell-based testing. The HepaRG™ genetic toxicology assay is being developed with support from ILS internal funding and NIEHS SBIR 4R44ES024698-02. The remaining authors declare that the research was conducted in the absence of any commercial or financial relationships that could be construed as a potential conflict of interest.

## Publisher's Note

All claims expressed in this article are solely those of the authors and do not necessarily represent those of their affiliated organizations, or those of the publisher, the editors and the reviewers. Any product that may be evaluated in this article, or claim that may be made by its manufacturer, is not guaranteed or endorsed by the publisher.

## Abbreviations

AFB1: Aflatoxin B1
BaP: Benzo[a]pyrene
CISP: Cisplatin
CP: Cyclophosphamide
AraC: Cytosine Arabinoside
DDI: DNA damage-inducing
2DG: 2-Deoxy-D-Glucose
HC: hierarchical clustering
MMS: Methyl methanesulfonate
ENU: N-Nitroso-N-Ethylurea
EUG: Eugenol
PG: Propyl Gallate
ZDV: Zidovudine
HTTr: High-Throughput Transcriptomics
MN: Micronuclei
MoA: Mode of Action
NAM: New Approach Methodology
non-DDI: non-DNA damage-inducing
NSC: Nearest Shrunken Centroids
PCA: Principal Component Analysis
SSBs: Single Strand Breaks
TGx: toxicogenomics.
