# Supplementary material for: A Modern Genotoxicity Testing Paradigm: Integration of the High-Throughput CometChip® and the TGx-DDI Transcriptomic Biomarker in Human HepaRG™ Cell Cultures
Source: Front Public Health. 2021 Aug 18;9:694834. doi: 10.3389/fpubh.2021.694834 (PMC8416458; doi:10.3389/fpubh.2021.694834)
Supplement: Supplementary file 1 [file Data_Sheet_1.PDF]

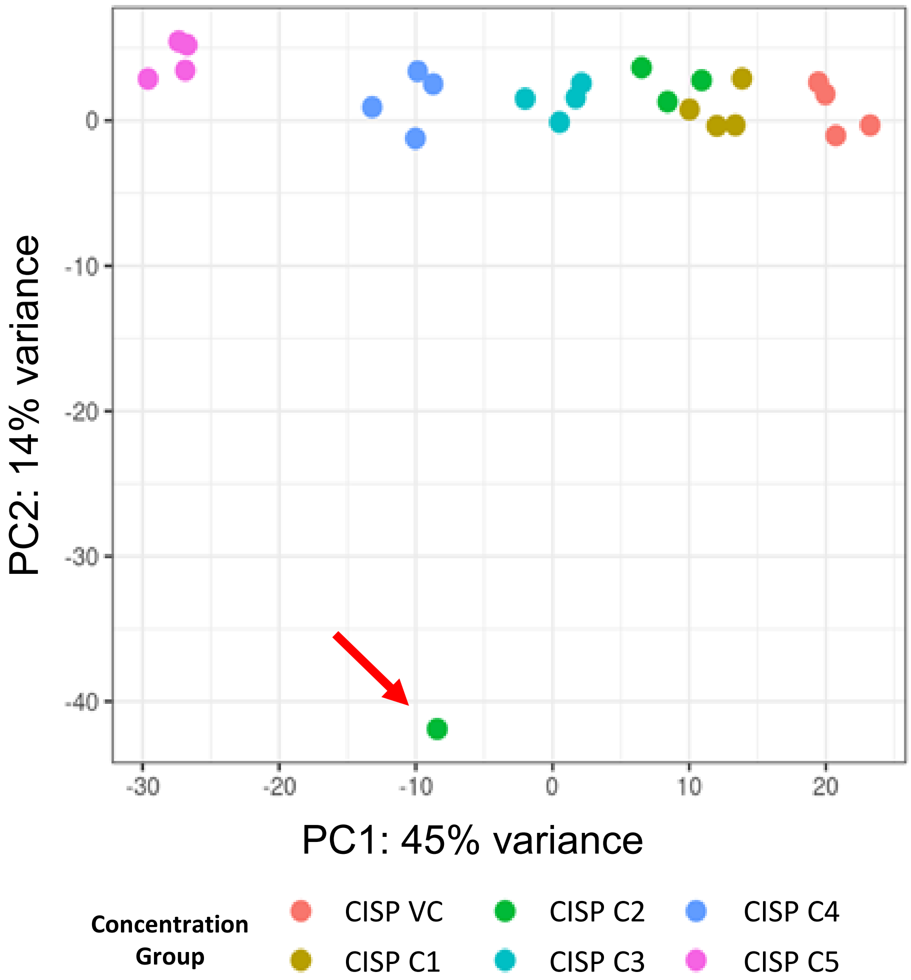

Supplementary Figure 1. Principal Component Analysis (PCA) of control samples and samples exposed to cisplatin (CISP) at five different concentrations for outlier identification and removal. Concentrations of CISP used for exposure in HepaRG™ cell cultures: C1 = 3.125 µM, C2 = 6.25 µM, C3 = 12.5 µM, C4 = 25 µM, and C5 = 50 µM (4 replicates per concentration group). PC1 = Principal Component 1 and PC2 = Principal Component 2. One replicate of CISP C2 was identified as an outlier, which is indicated with a red arrow.

Supplementary Table 1: HepaRG™ cell viability measured using the CellTiter-Glo® Luminescent Cell Viability Assay

	**Corresponding Cell Viability (as a % compared to Vehicle Control)**				
**Group 1 Chemicals**	**C1**	**C2**	**C3**	**C4**	**C5**
Aflatoxin B1	107	101	90	97	67
Benzo[a]pyrene	98	113	103	67	50
Cisplatin	127	134	119	125	110
Cyclophosphamide	100	100	93	68	57
Cytosine Arabinoside	94	93	90	83	79
Methyl Methanesulfonate	103	105	108	88	80
N-Ethyl-N-Nitrosourea	99	101	89	70	**3**
Zidovudine (Azidothymidine)	118	116	121	113	118
Propyl Gallate	88	106	71	41	**18**
**Group 2 Chemicals**	**C1**	**C2**	**C3**	**C4**	**C5**
2-Deoxy-D-Glucose	115	116	120	104	98
**Group 3 Chemicals**	**C1**	**C2**	**C3**	**C4**	**C5**
Eugenol	104	107	100	56	**0**
Urea	109	106	113	113	109

C1 (lowest concentration tested) to C5 (highest concentration tested). Cytotoxic cut-off is > 60% cytotoxic (corresponds to < 40% cell viability). Bolded Cell Viability indicates that the corresponding concentration has surpassed the cytotoxicity threshold.

Supplementary Figure 2A: Aflatoxin B1 (AFB1)

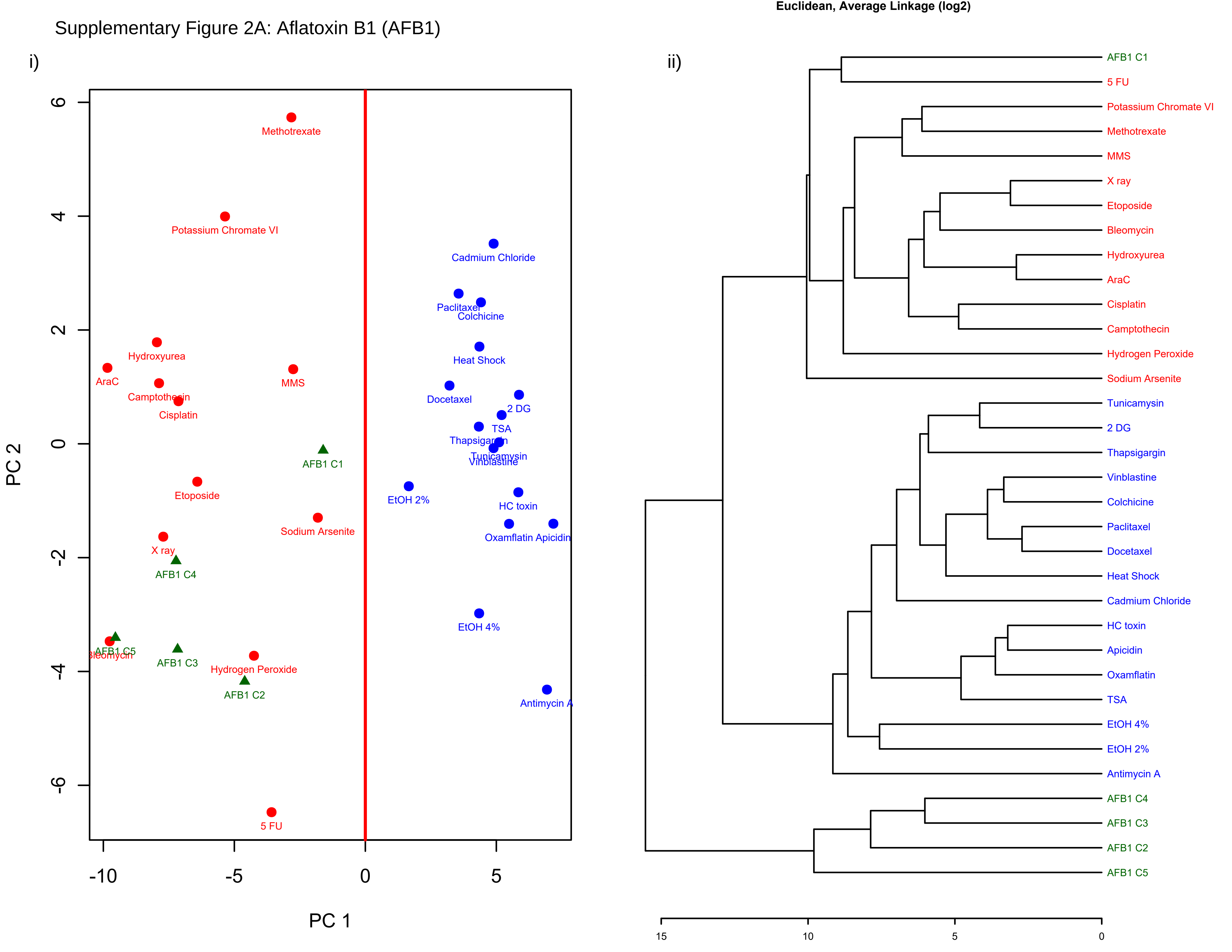

Supplementary Figure 2B: Benzo[a]pyrene (BaP)

Euclidean, Average Linkage (log2)

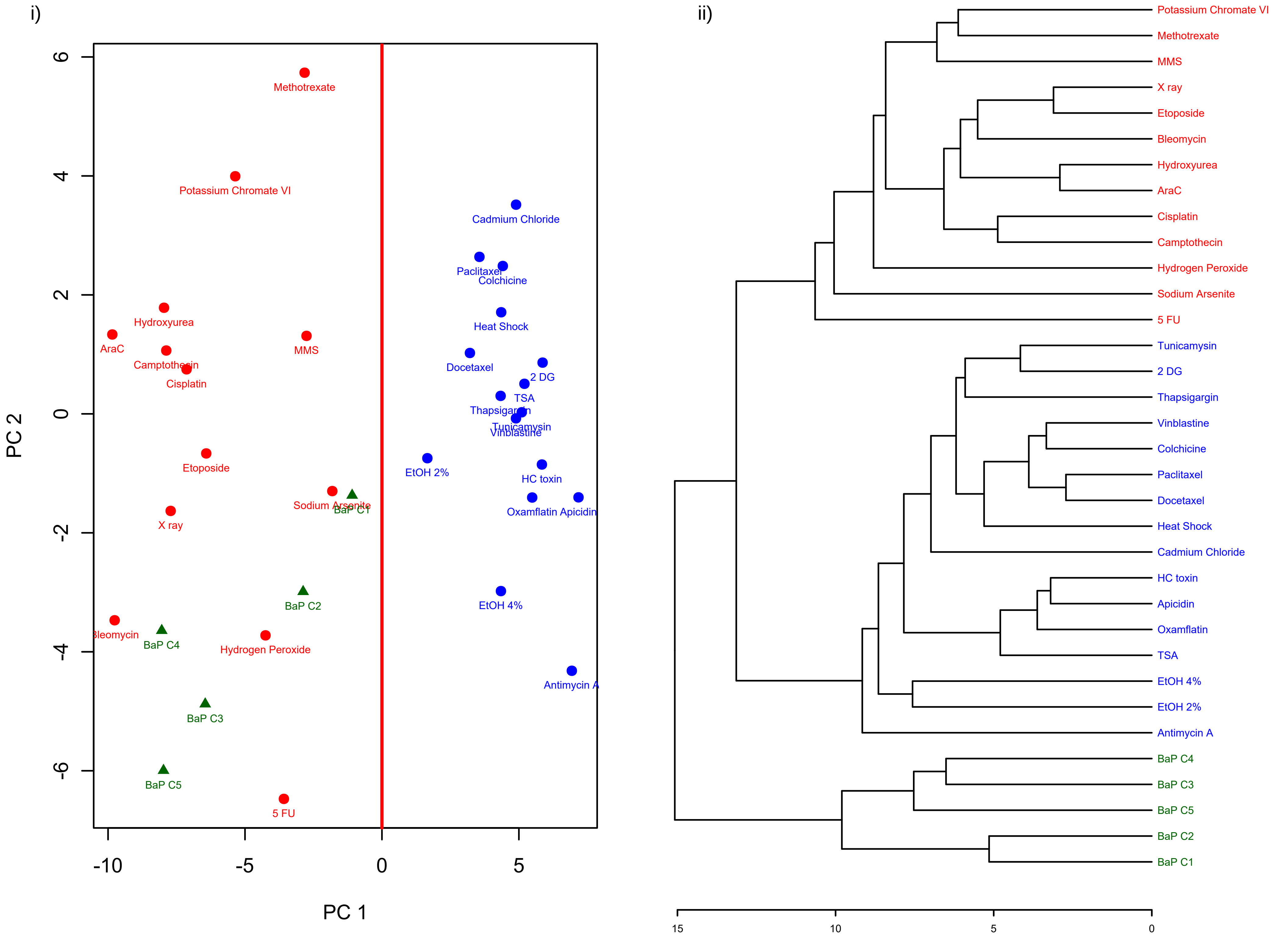

Supplementary Figure 2C: Cisplatin (CISP)

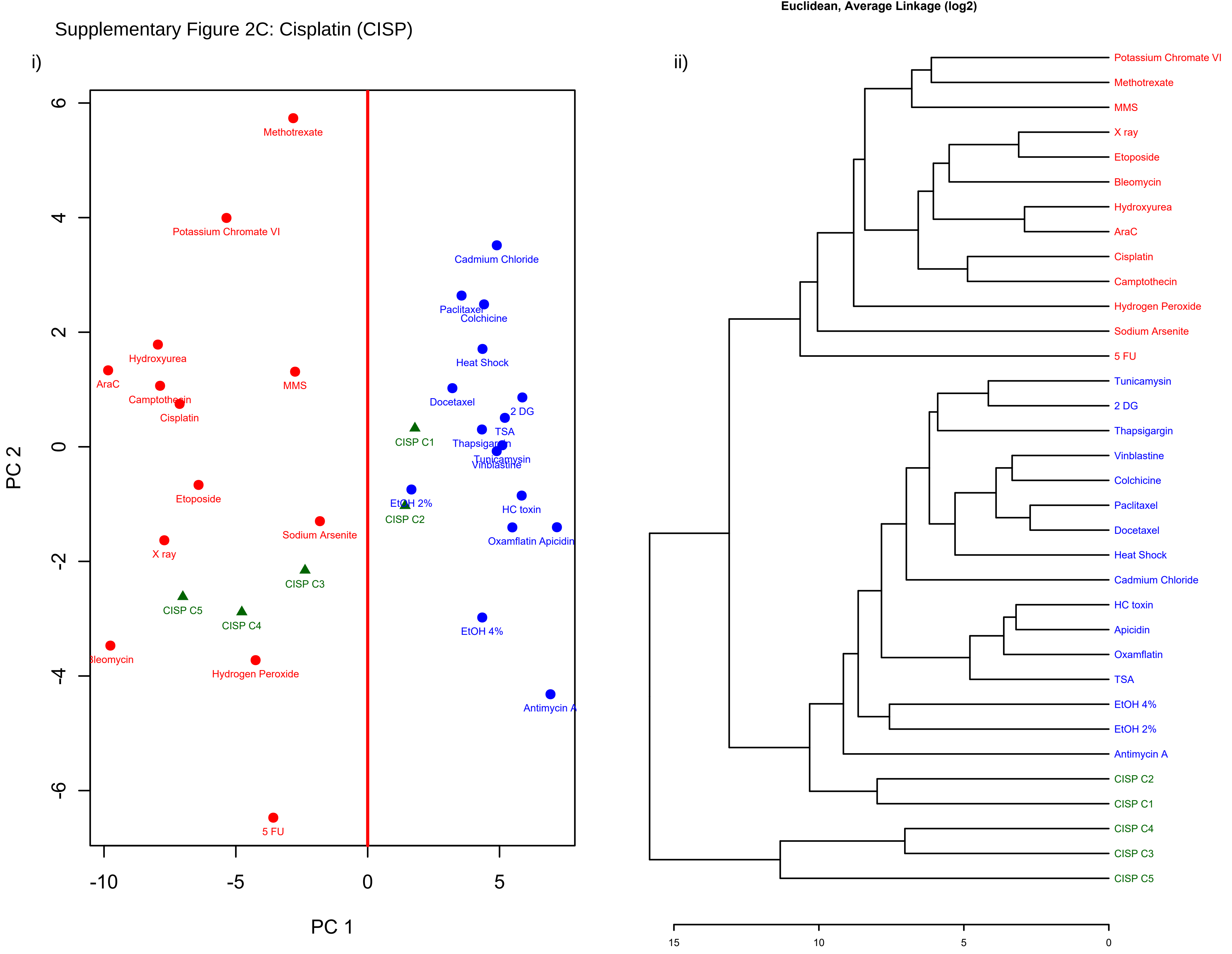

Supplementary Figure 2D: Cyclophosphamide (CP)

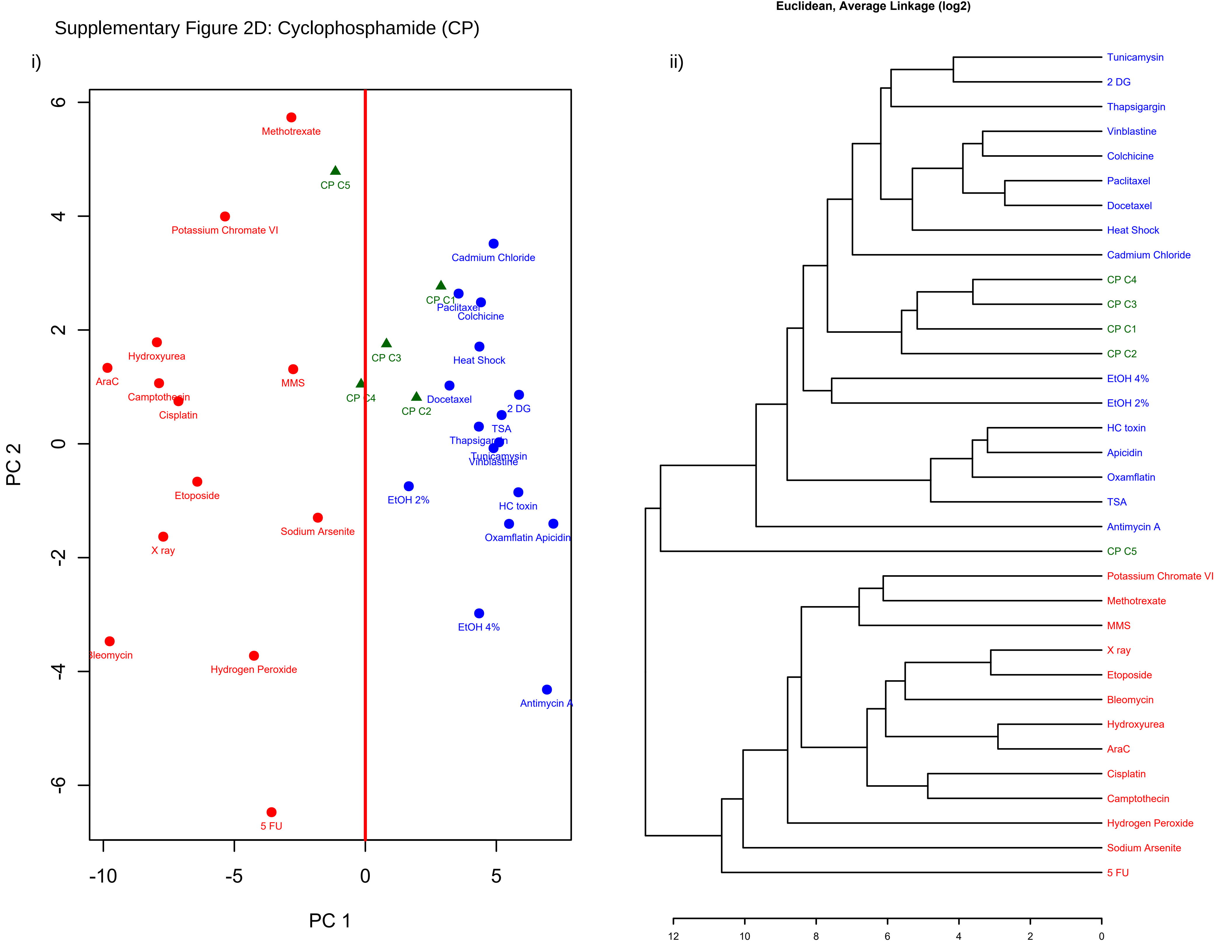

Supplementary Figure 2E: Cytosine Arabinoside (AraC)

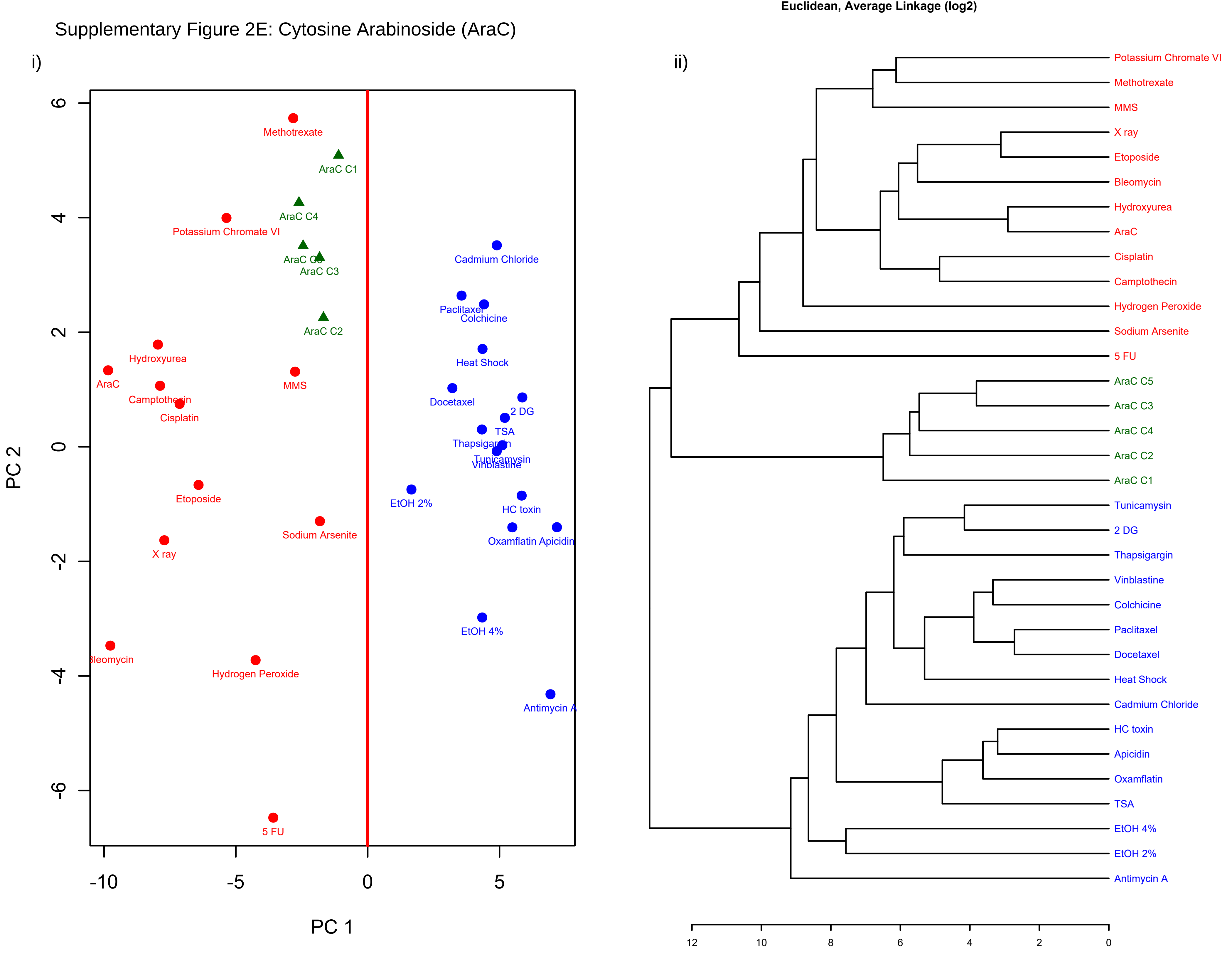

Supplementary Figure 2F: Methyl Methanesulfonate (MMS)

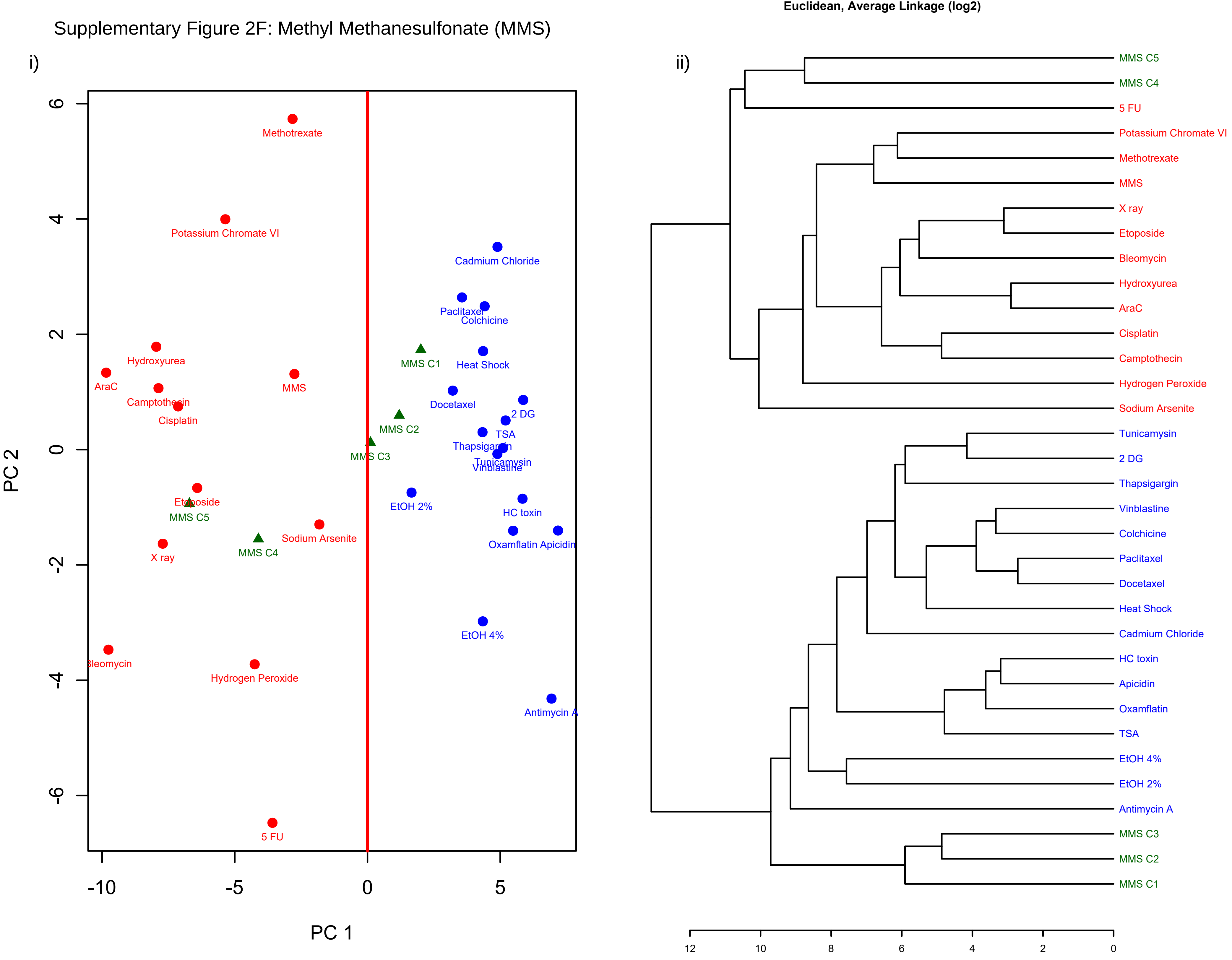

Supplementary Figure 2G: N-Nitroso-N-Ethylurea (ENU)

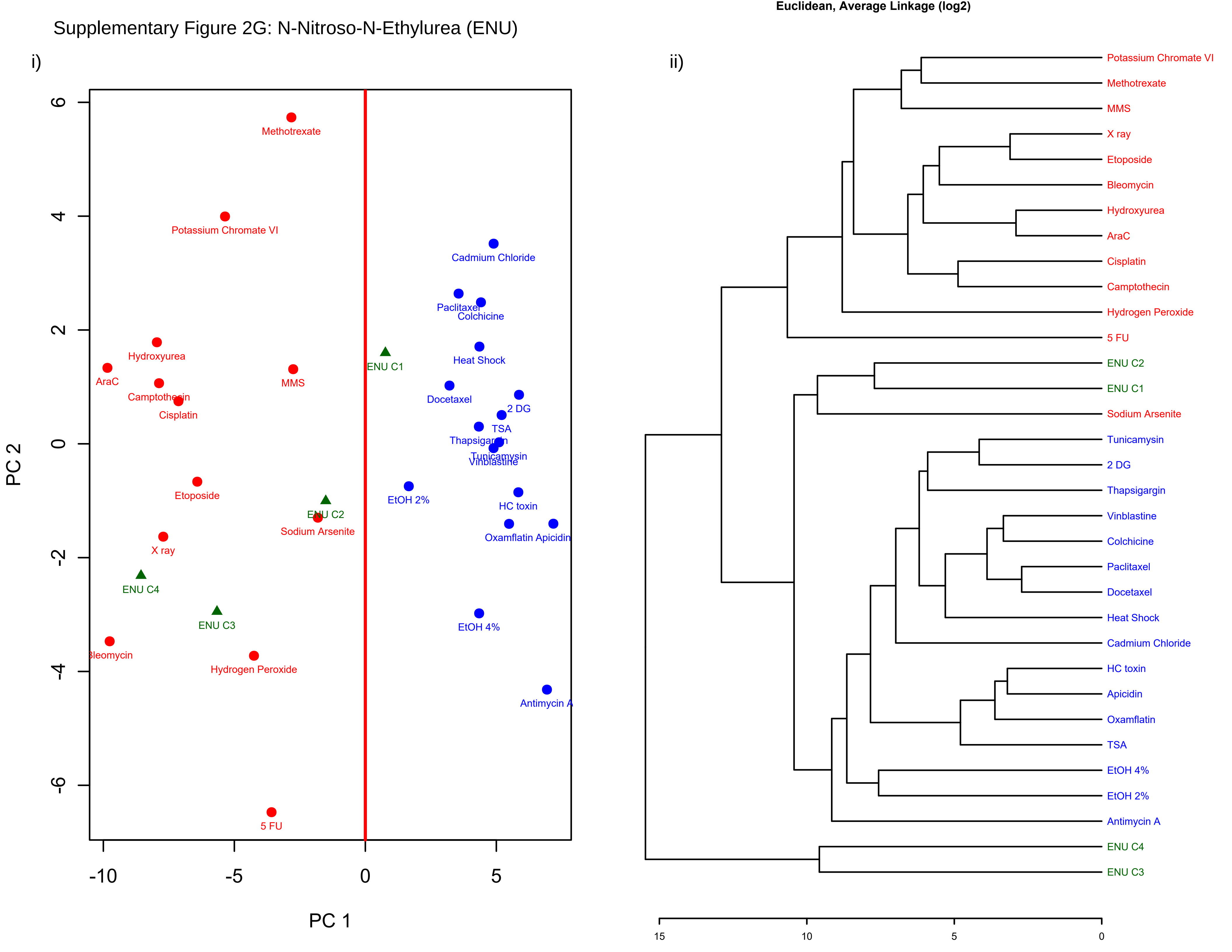

Supplementary Figure 2H: Zidovudine (ZDV)

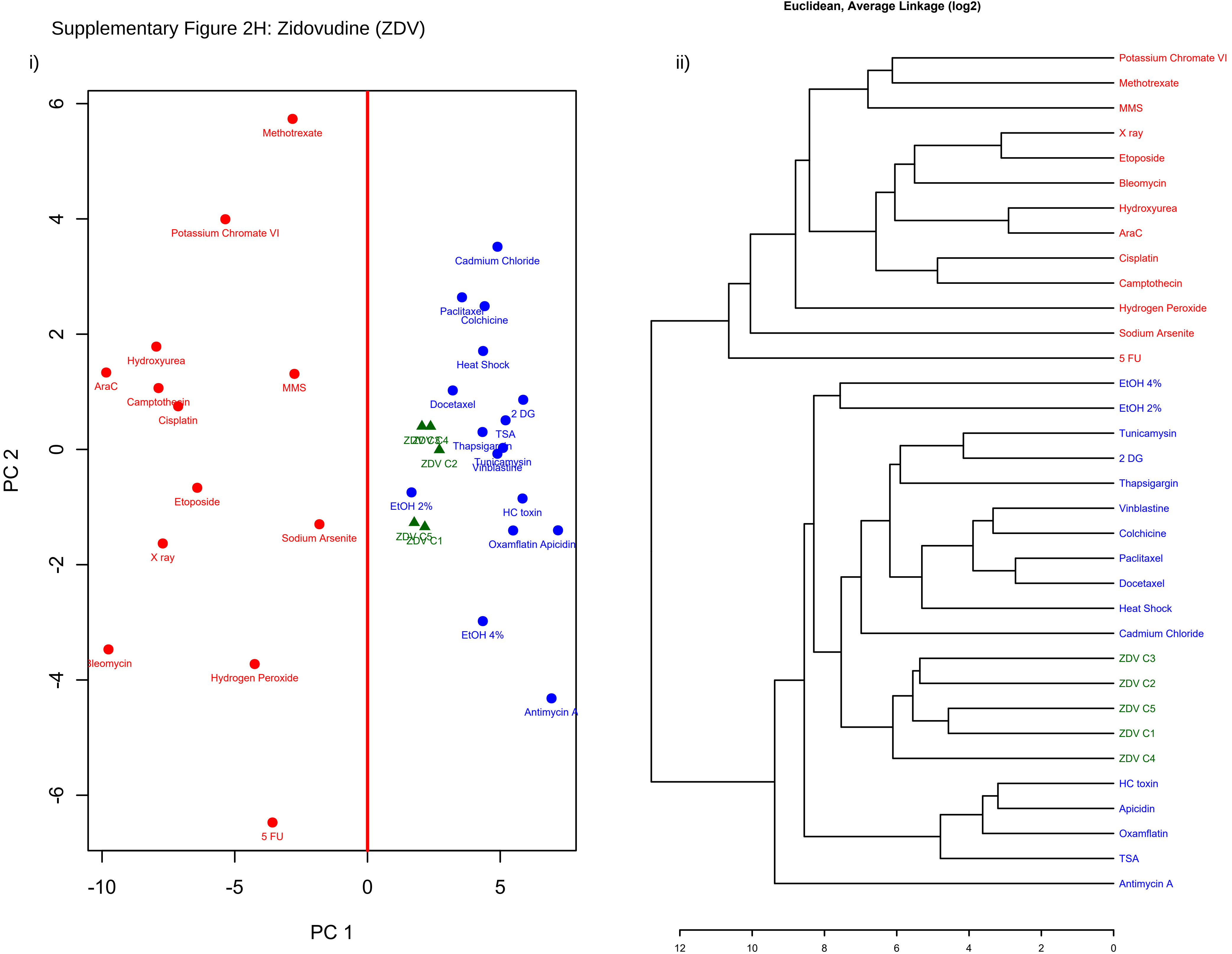

Supplementary Figure 2I: Propyl Gallate (PG)

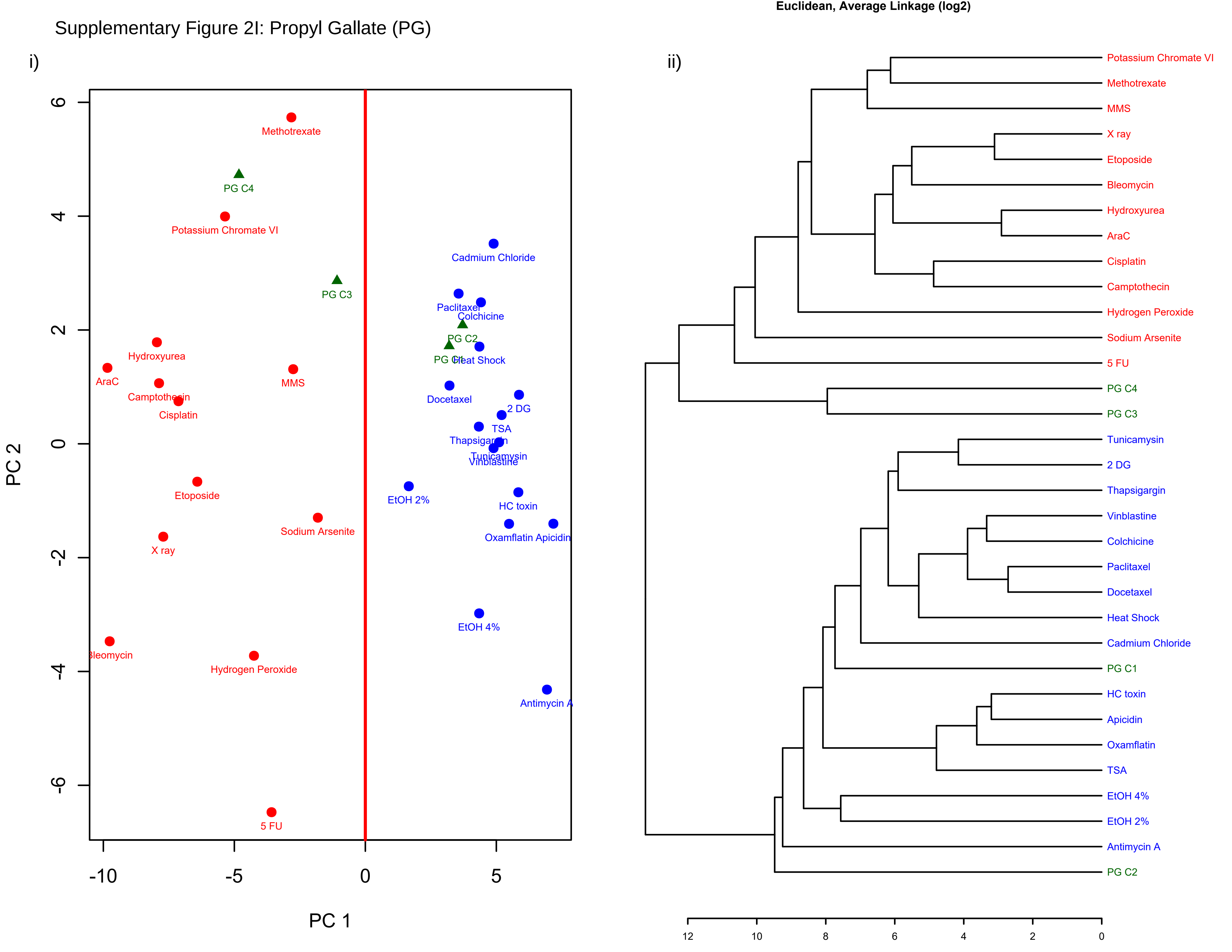

Supplementary Figure 2J: 2-Deoxy-D-Glucose (2DG)

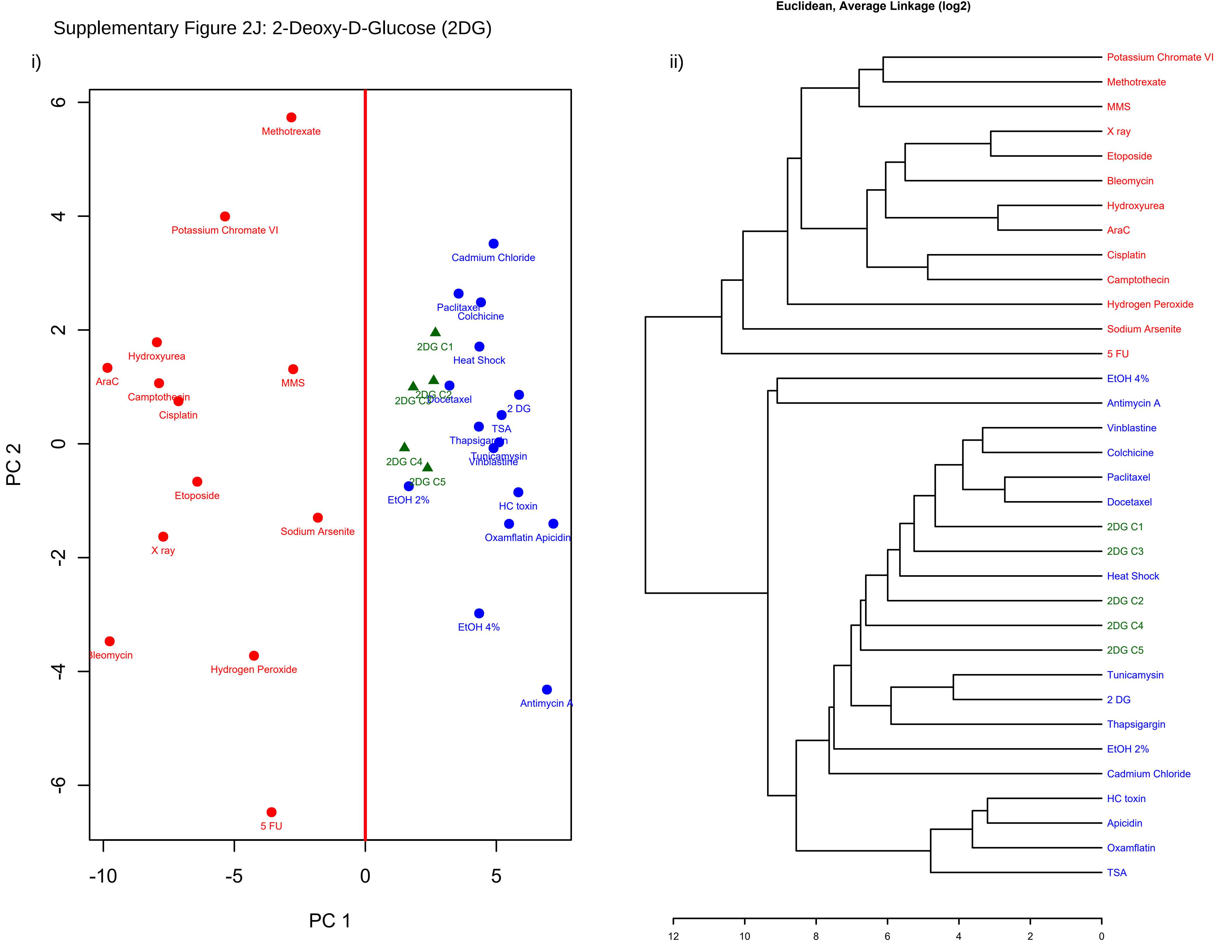

Supplementary Figure 2K: Eugenol (EUG)

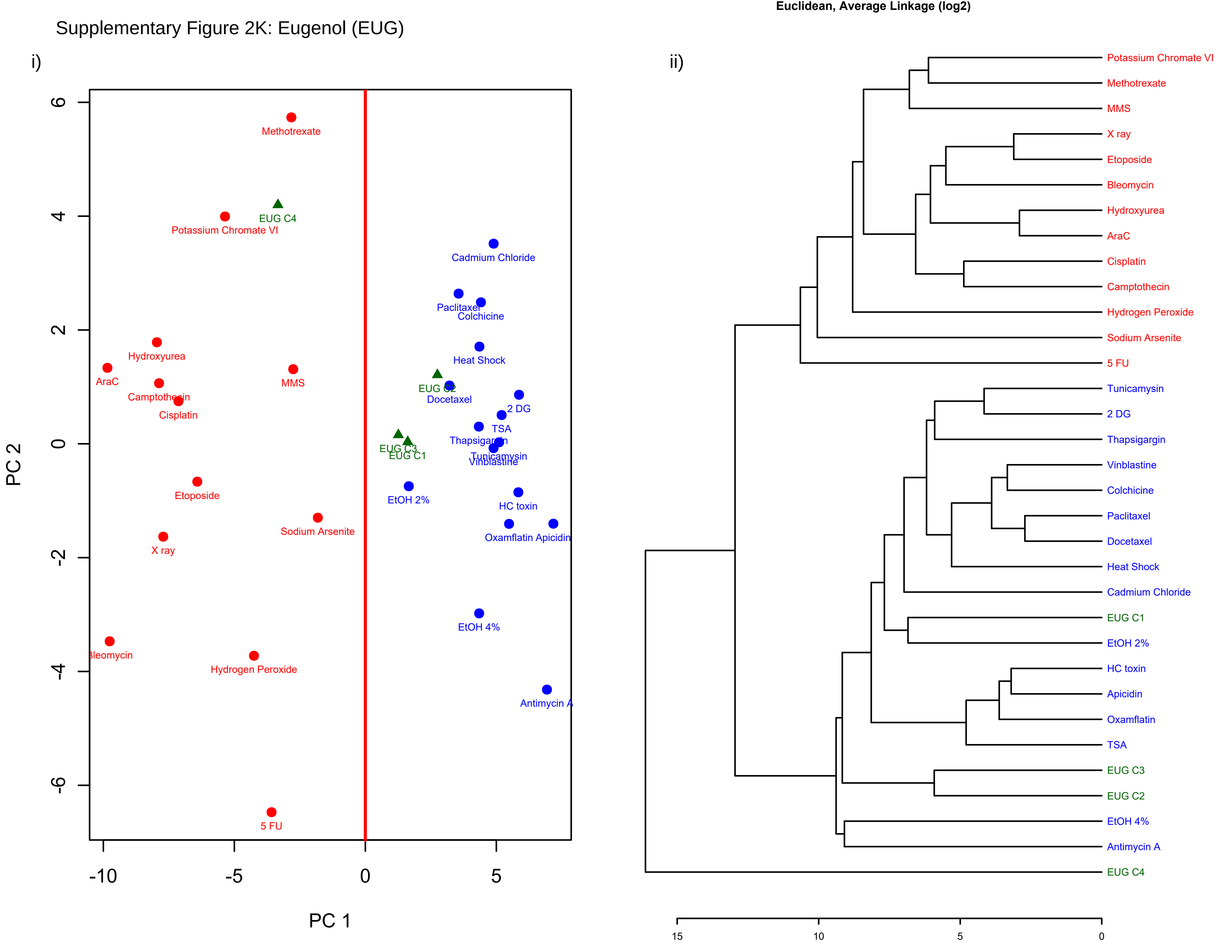

Supplementary Figure 2L: Urea

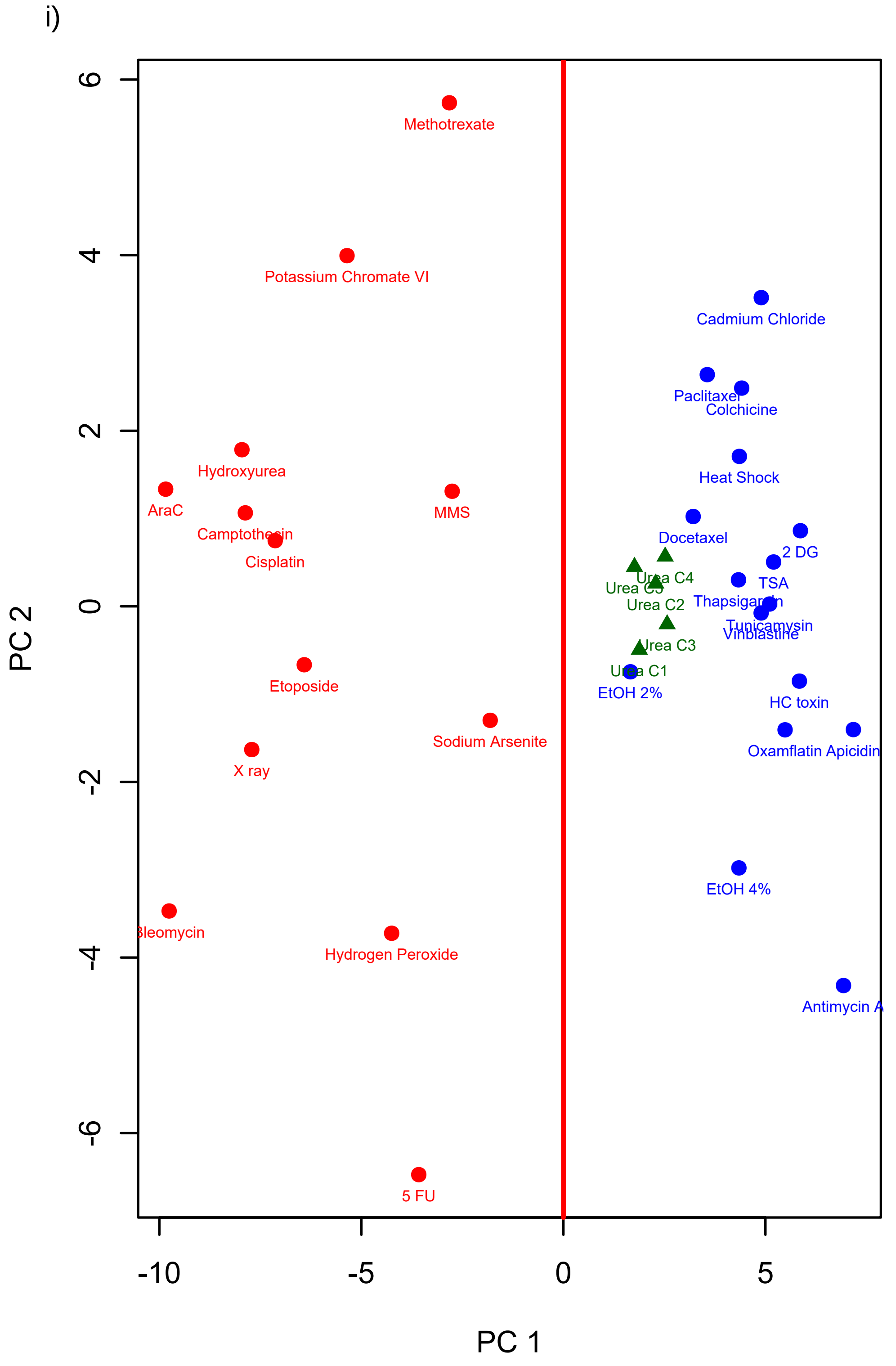

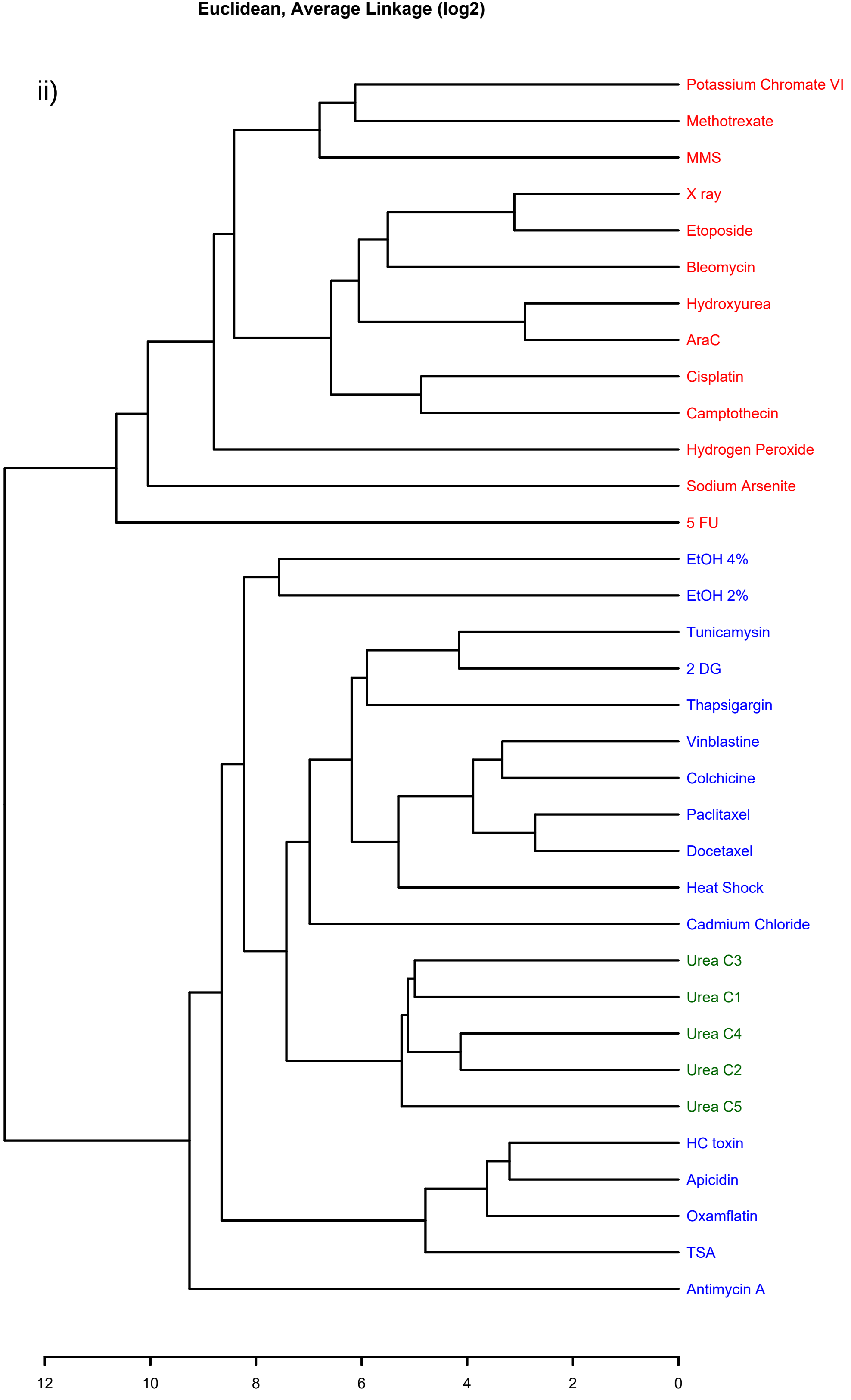

Supplementary Figure 2A-2L: Principal component analysis (PCA) and hierarchical clustering (HC) depicting TGx-DDI classification results for 12 test chemicals. DDI chemicals are shown in panels: (1A) aflatoxin B1 (AFB1), (1B) benzo[a]pyrene (BaP), (1C) cisplatin (CISP), (1D) cyclophosphamide (CP), (1E) cytosine arabinoside (AraC), (1F) methyl methanesulfonate (MMS), (1G) N-ethyl-N-nitrosourea (ENU), (1H) zidovudine (ZVD), and (1I) propyl gallate (PG). The non-DDI chemical and misleading positive chemicals are depicted in panels: (1J) 2-deoxy-D-glucose (2DG), (1K) eugenol (EUG), and (1L) urea. PCA using the TGx-DDI biomarker for TK6 cells exposed to the training set of chemicals (red font = DDI training set; blue font = non-DDI training set) and for human HepaRG™ cells exposed to 12 test chemicals at five increasing concentrations (C1-C5) 4 hr after the last exposure (green font = replicates of test agent) are shown on the left side of the figure for each chemical (panel i). The line drawn at 0 on the PCA plot divides the DDI and non-DDI agents and was used for classification. Hierarchical clustering of the chemicals based on TGx-DDI classification analysis are shown on the right side of figure for each chemical (panel ii), with color-coding as indicated for PCA. The main branch on the dendrogram separates the DDI and non-DDI agents and was used for classification of the test agent.
